# Supplementary material for: Dynamic modulation of modal coupling in microelectromechanical gyroscopic ring resonators
Source: Nat Commun. 2019 Oct 31;10:4980. doi: 10.1038/s41467-019-12796-0 (PMC6823415; doi:10.1038/s41467-019-12796-0)
Supplement: Supplementary file 1 — Supplementary Information [file 41467_2019_12796_MOESM1_ESM.pdf]

Dynamic Modulation of Modal Coupling in Microelectromechanical Gyroscopic Ring Resonators

Zhou et al.

Supplementary Note 1. The ring resonator and degenerate wineglass modes

The top view false-colour SEM picture of the ring resonator structure before encapsulating is shown in Figure **1**a. The orange part is the resonator structure, and the blue parts are capacitive electrodes. A cross-section view of the resonator is provided in Figure **1**b. The resonator is fabricated using a SOI wafer. The resonator and electrodes are patterned and separated by slots etched by deep reactive ion etching process. The flexure nested rings are released by etching the SiO_2 layer underneath. This "Epi-seal" process used to fabricate the devices is described in detail in ref. [1].

The in-plane deformation of the ideal ring structure can be expressed by the radial displacement $W(\phi, t)$ and tangential displacement $V(\phi, t)$ of its mid surface, both of which are functions of circumferential position ϕ and time t. The displacements of the n-th degenerate in-plane modes are given by $W_{n1}(\phi, t) = W_{1,0}\cos(n\phi)\cos(\omega_n t)$, $V_{n1}(\phi, t) = V_{1,0}\sin(n\phi)\cos(\omega_n t)$, and $W_{n2}(\phi, t) = W_{2,0}\sin(n\phi)\cos(\omega_n t)$, $V_{n2}(\phi, t) = V_{2,0}\cos(n\phi)\cos(\omega_n t)$. Thus, the antinodal axes of the n-th degenerate modes have an angular interval of $90°/n$. The order-2 degenerate modes with antinodal axes interval of 45° can be equivalent to a reduced-order two-degree-of-freedom lumped parameter system, in which the antinodal axes interval is 90°. The angle in the equivalent coordinates is the double of that in the real setup coordinates. In this paper, unless otherwise specified, all the specified angles are based on equivalent coordinates.

Supplementary Note 2. Structural asymmetry hybrid state coupling and electrostatic tuning

The relationship between coordinates x-o-y and x_ω-o-y_ω is

$$\begin{bmatrix} x_\omega \\ y_\omega \end{bmatrix} = \underbrace{\begin{bmatrix} \cos\theta & \sin\theta \\ -\sin\theta & \cos\theta \end{bmatrix}}_{\mathbf{p}} \begin{bmatrix} x \\ y \end{bmatrix}. \tag{1}$$

The homogeneous equations of motion (EOMs) of the two normal modes II-1 and II-2 oscillating along x_ω-o-y_ω are given by

$$\begin{bmatrix} \ddot{x}_\omega \\ \ddot{y}_\omega \end{bmatrix} + \gamma_{\text{II}} \begin{bmatrix} \dot{x}_\omega \\ \dot{y}_\omega \end{bmatrix} + \begin{bmatrix} \omega_{\text{II-1}}^2 & 0 \\ 0 & \omega_{\text{II-2}}^2 \end{bmatrix} \begin{bmatrix} x_\omega \\ y_\omega \end{bmatrix} = \mathbf{0}. \tag{2}$$

Since the drive, pump, and tuning signal are applied along x-o-y, we transform the EOMs into x-o-y using coordinate-transformation matrix $\mathbf{p}$,

$$\begin{bmatrix} \ddot{x} \\ \ddot{y} \end{bmatrix} + \gamma_{\text{II}} \begin{bmatrix} \dot{x} \\ \dot{y} \end{bmatrix} + \mathbf{p}^{-1} \begin{bmatrix} \omega_{\text{II-1}}^2 & 0 \\ 0 & \omega_{\text{II-2}}^2 \end{bmatrix} \mathbf{p} \begin{bmatrix} x \\ y \end{bmatrix} = \mathbf{0}. \tag{3}$$

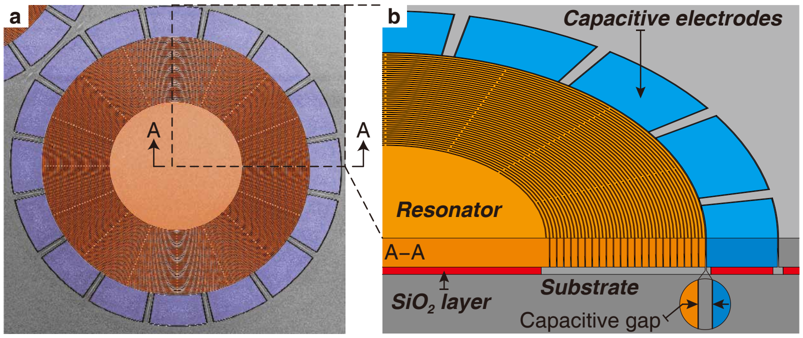

Supplementary Figure 1. Description of the ring resonator. **a** Top view SEM picture of the ring resonator. **b** Schematic of the cross-section A-A marked in (**a**), a quarter of the resonator is shown.

Further introducing drive into EOMs leads to

$$\begin{bmatrix} \ddot{x} \\ \ddot{y} \end{bmatrix} + \gamma_{\text{II}} \begin{bmatrix} \dot{x} \\ \dot{y} \end{bmatrix} + \begin{bmatrix} \omega_{\text{II-1}}^2 \cos^2\theta + \omega_{\text{II-2}}^2 \sin^2\theta & (\omega_{\text{II-1}}^2 - \omega_{\text{II-2}}^2)\cos\theta\sin\theta \\ (\omega_{\text{II-1}}^2 - \omega_{\text{II-2}}^2)\cos\theta\sin\theta & \omega_{\text{II-1}}^2 \sin^2\theta + \omega_{\text{II-2}}^2 \cos^2\theta \end{bmatrix} \begin{bmatrix} x \\ y \end{bmatrix} = \begin{bmatrix} F\cos\omega_{\text{d}}t \\ 0 \end{bmatrix}, \quad (4)$$

where $F\cos\omega_{\text{d}}t$ is the mass normalized driving force induced by $\pm V_{\text{d}}\cos(\omega_{\text{d}}t)$ in push-pull form,

$$F\cos\omega_{\text{d}}t = \frac{A_{\text{d}}\epsilon_0[V_0 + V_{\text{d}}\cos\omega_{\text{d}}t]^2}{2d_0^2 m_{\text{II}}} - \frac{A_{\text{d}}\epsilon_0[V_0 - V_{\text{d}}\cos\omega_{\text{d}}t]^2}{2d_0^2 m_{\text{II}}} \approx \frac{2A_{\text{d}}\epsilon_0 V_{\text{d}} V_0}{d_0^2 m_{\text{II}}}\cos\omega_{\text{d}}t, \quad (5)$$

A_{d} is the area of single drive electrode, and m_{II} is the effective mass of the $n = 2$ normal modes. The normalized stiffness matrix in equation (4) is nondiagonal, which indicates that states detected along x and y directions are coupled. Those two hybrid states are combinations of normal modes II-1 and II-2.

The schematic of electrostatic tuning by varying V_{t1} is depicted by Figure **2**a. The stiffness of each modes is affected by the DC voltage V_0 before the tuning voltage is applied. The resonant frequencies of the bare mechanical resonators excluding the V_0 influence are $\omega_{\square}^* = \sqrt{\omega_{\square}^2 + A_{\text{t}}\epsilon_0 V_0^2/(d_0^3 m_{\text{II}})}$, where $\square = $ II-1 or II-2, A_{t} is the area of tuning electrode. The tuning is applied along x direction. The system can be equivalent to introducing a stiffness (normalized by mass m_{II}) $\Delta_{\text{t1}} = A_{\text{t}}\epsilon_0 V_{\text{t1}}(2V_0 - V_{\text{t1}})/(d_0^3 m_{\text{II}})$ along x direction to original system.

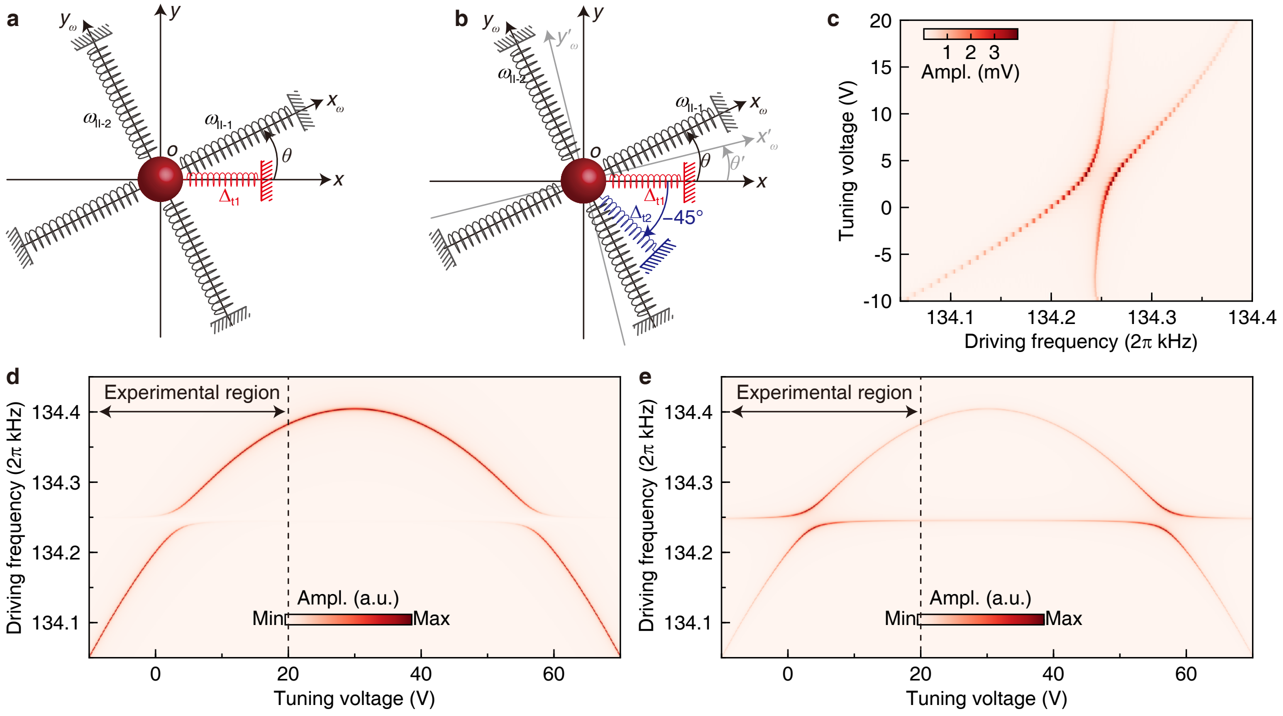

Supplementary Figure 2. **a** Schematic of electrostatic tuning by applying V_{t1} along x axis. **b** Both V_{t1} along x axis and V_{t2} along $-45°$ axis can affect θ. **c** Frequency responses detected along y axis at ω_{d} with different values of V_{t1}. **d**, **e** Simulation of frequency responses with different values of V_{t1} along x and y axes, respectively. Source data are provided as a Source Data file.

Thus, the V_{t1} tuning process can be described by the following equations,

$$\ddot{x} + \gamma_{\text{II}}\dot{x} + \Bigg[\underbrace{\omega_{\text{II-1}}^2\cos^2\theta + \omega_{\text{II-2}}^2\sin^2\theta}_{\Omega_1} + \underbrace{\frac{A_{\text{t}}\epsilon_0 V_{\text{t1}}(2V_0 - V_{\text{t1}})}{d_0^3 m_{\text{II}}}}_{\Delta_{\text{t1}}}\Bigg]x + \underbrace{(\omega_{\text{II-1}}^2 - \omega_{\text{II-2}}^2)\cos\theta\sin\theta}_{\Pi}\, y$$

$$= \underbrace{\frac{2A_{\text{d}}\epsilon_0 V_{\text{d}} V_0}{d_0^2 m_{\text{II}}}}_{F}\cos\omega_{\text{d}}t, \tag{6}$$

$$\ddot{y} + \gamma_{\text{II}}\dot{y} + \underbrace{(\omega_{\text{II-1}}^2\sin^2\theta + \omega_{\text{II-2}}^2\cos^2\theta)}_{\Omega_2}\, y + \underbrace{(\omega_{\text{II-1}}^2 - \omega_{\text{II-2}}^2)\cos\theta\sin\theta}_{\Pi}\, x = 0. \tag{7}$$

The V_{t1} tuning process can be simulated by numerically solving above equations using slowly varying envelope approximation [2]. By introducing slowly varying complex amplitudes $A(t)$ and $B(t)$, the displacements in equations (6,7) can be approximated by $x = A(t)e^{i\omega_{\text{d}}t} + cc$ and $y = B(t)e^{i\omega_{\text{d}}t} + cc$, where cc denotes the complex conjugate of the preceding terms. Then the equations (6,7) can be

rewritten as

$$(i\omega_\mathrm{d} + \gamma_\mathrm{II})\dot{A} + (i\omega_\mathrm{d}\gamma_\mathrm{II} - \omega_\mathrm{d}^2 + \Omega_1 + \Delta_\mathrm{t1})A + \Pi B = \frac{F}{2}, \tag{8}$$

$$(i\omega_\mathrm{d} + \gamma_\mathrm{II})\dot{B} + (i\omega_\mathrm{d}\gamma_\mathrm{II} - \omega_\mathrm{d}^2 + \Omega_2)B + \Pi A = 0. \tag{9}$$

The second time derivative of the complex amplitudes have been neglected. For the steady-state analysis, $\dot{A} = \dot{B} = 0$. The complex amplitudes can be approximated to be

$$A = \frac{-F(i\omega_\mathrm{d}\gamma_\mathrm{II} - \omega_\mathrm{d}^2 + \Omega_2)}{2(\Pi^2 - (i\omega_\mathrm{d}\gamma_\mathrm{II} - \omega_\mathrm{d}^2 + \Omega_1 + \Delta_\mathrm{t1})(i\omega_\mathrm{d}\gamma_\mathrm{II} - \omega_\mathrm{d}^2 + \Omega_2))}, \tag{10}$$

$$B = \frac{F\Pi}{2(\Pi^2 - (i\omega_\mathrm{d}\gamma_\mathrm{II} - \omega_\mathrm{d}^2 + \Omega_1 + \Delta_\mathrm{t1})(i\omega_\mathrm{d}\gamma_\mathrm{II} - \omega_\mathrm{d}^2 + \Omega_2))}. \tag{11}$$

Frequency responses can be obtained by calculating the frequency-dependent amplitudes $|A|$ and $|B|$ (Fig. **2**d,e).

The misalignment θ can be adjusted by tuning voltages V_t1 and V_t2. Wherein V_t2 is applied along $-45°$ off-axis electrodes, which can be equivalent to introducing a stiffness (normalized by mass m_II) $\Delta_\mathrm{t2} = A_\mathrm{t}\epsilon_0 V_\mathrm{t2}(2V_0 - V_\mathrm{t2})/(d_0^3 m_\mathrm{II})$ along $-45°$ off-axis direction to original system (Fig. **2**b). If both V_t2 and V_t2 are applied, the primary orientation of normal modes change to x'_ω-o-y'_ω, the misalignment of new normal modes with electrode axes become θ'. The stiffness matrix of the tuned system along x'_ω-o-y'_ω is given by

$$\mathbf{M}_\mathrm{stiffness} = \mathbf{t}^{-1}\begin{bmatrix} \omega_\text{II-1}^2 & 0 \\ 0 & \omega_\text{II-2}^2 \end{bmatrix}\mathbf{t} + \mathbf{s}^{-1}\begin{bmatrix} \Delta_\mathrm{t2} & 0 \\ 0 & 0 \end{bmatrix}\mathbf{s} + \mathbf{r}^{-1}\begin{bmatrix} \Delta_\mathrm{t1} & 0 \\ 0 & 0 \end{bmatrix}\mathbf{r}, \tag{12}$$

where

$$\mathbf{t} = \begin{bmatrix} \cos(\theta - \theta') & \sin(\theta - \theta') \\ -\sin(\theta - \theta') & \cos(\theta - \theta') \end{bmatrix}, \tag{13}$$

$$\mathbf{s} = \begin{bmatrix} \cos(-45° - \theta') & \sin(-45° - \theta') \\ -\sin(-45° - \theta') & \cos(-45° - \theta') \end{bmatrix}, \tag{14}$$

$$\mathbf{r} = \begin{bmatrix} \cos\theta' & -\sin\theta' \\ \sin\theta' & \cos\theta' \end{bmatrix}. \tag{15}$$

By using the condition of $\mathbf{M}_\mathrm{stiffness}$ being diagonal, we obtain

$$(\omega_\text{II-1}^2 - \omega_\text{II-2}^2)\cos(\theta - \theta')\sin(\theta - \theta') + \Delta_\mathrm{t2}\sin(-45° - \theta')\cos(-45° - \theta') - \Delta_\mathrm{t1}\sin\theta'\cos\theta' = 0. \tag{16}$$

Thus, the adjusted θ' by applying tuning voltages V_{t1} and V_{t2} is given by

$$\theta' = \frac{1}{2}\arctan\frac{(\omega_{\text{II-1}}^2 - \omega_{\text{II-2}}^2)\sin(2\theta) - \Delta_{t2}}{(\omega_{\text{II-1}}^2 - \omega_{\text{II-2}}^2)\cos(2\theta) + \Delta_{t1}}. \tag{17}$$

V_{t1} and V_{t2} may also influence the eigenfrequencies $\omega_{\text{II-1}}$ and $\omega_{\text{II-2}}$. The stiffness matrix of the tuned system along x-o-y is given by

$$\mathbf{M}'_{\text{stiffness}} = \mathbf{p}^{-1}\begin{bmatrix}\omega_{\text{II-1}}^2 & 0\\ 0 & \omega_{\text{II-2}}^2\end{bmatrix}\mathbf{p} + \begin{bmatrix}\Delta_{t1} & 0\\ 0 & 0\end{bmatrix} + \mathbf{q}^{-1}\begin{bmatrix}\Delta_{t2} & 0\\ 0 & 0\end{bmatrix}\mathbf{q}, \tag{18}$$

where

$$\mathbf{q} = \begin{bmatrix}\cos(45°) & -\sin(45°)\\ \sin(45°) & \cos(45°)\end{bmatrix}. \tag{19}$$

Calculating the eigenvalues $\lambda'_\pm$ of $\mathbf{M}'_{\text{stiffness}}$ by solving $\det(\mathbf{M}'_{\text{stiffness}} - \lambda'_\pm\mathbf{I}) = 0$, we obtain the tuned eigenfrequencies,

$$\begin{aligned}\omega'^2_{\text{II-1}} \equiv \lambda'_- = {} & \frac{\omega_{\text{II-1}}^2 + \omega_{\text{II-2}}^2 + \Delta_{t1} + \Delta_{t2}}{2}\\ & - \frac{1}{2}\sqrt{\left[\omega_{\text{II-1}}^2 - \omega_{\text{II-2}}^2 + \Delta_{t1}\cos(2\theta) - \Delta_{t2}\sin(2\theta)\right]^2 + \left[\Delta_{t1}\sin(2\theta) + \Delta_{t2}\cos(2\theta)\right]^2},\end{aligned} \tag{20}$$

$$\begin{aligned}\omega'^2_{\text{II-2}} \equiv \lambda'_+ = {} & \frac{\omega_{\text{II-1}}^2 + \omega_{\text{II-2}}^2 + \Delta_{t1} + \Delta_{t2}}{2}\\ & + \frac{1}{2}\sqrt{\left[\omega_{\text{II-1}}^2 - \omega_{\text{II-2}}^2 + \Delta_{t1}\cos(2\theta) - \Delta_{t2}\sin(2\theta)\right]^2 + \left[\Delta_{t1}\sin(2\theta) + \Delta_{t2}\cos(2\theta)\right]^2}.\end{aligned} \tag{21}$$

The tuned frequency difference $\Delta\omega' = \omega'_{\text{II-2}} - \omega'_{\text{II-1}}$ can be further obtained.

Supplementary Note 3. Dynamical sideband coupling based on hybrid state coupling

1. Dynamical sideband coupling model

Applying periodical signal $V_p\cos\omega_p t$ will cause complex modification of the stiffness along y axis. Similar to the applying of static tuning in Figure **2**a, a parametric pump Δ_p is introduced into equations (4),

$$\begin{aligned}&\begin{bmatrix}\ddot{x}\\ \ddot{y}\end{bmatrix} + \gamma_{\text{II}}\begin{bmatrix}\dot{x}\\ \dot{y}\end{bmatrix} + \begin{bmatrix}\omega_{\text{II-1}}^2\cos^2\theta + \omega_{\text{II-2}}^2\sin^2\theta & (\omega_{\text{II-1}}^2 - \omega_{\text{II-2}}^2)\cos\theta\sin\theta\\ (\omega_{\text{II-1}}^2 - \omega_{\text{II-2}}^2)\cos\theta\sin\theta & \omega_{\text{II-1}}^2\sin^2\theta + \omega_{\text{II-2}}^2\cos^2\theta + \Delta_p\end{bmatrix}\begin{bmatrix}x\\ y\end{bmatrix}\\ &= \begin{bmatrix}\frac{F\cos\omega_d t}{m_{\text{II}}}\\ 0\end{bmatrix},\end{aligned} \tag{22}$$

where

$$\Delta_{\mathrm{p}} = \underbrace{\frac{A_{\mathrm{p}}\epsilon_0}{d_0^3 m_{\mathrm{II}}}}_{\kappa}\left\{V_0^2 - [V_0 - V_{\mathrm{p}}\cos(\omega_{\mathrm{p}}t)]^2\right\} = \kappa\left[2V_0V_{\mathrm{p}}\cos(\omega_{\mathrm{p}}t) - \frac{V_{\mathrm{p}}^2}{2} - \frac{V_{\mathrm{p}}^2}{2}\cos(2\omega_{\mathrm{p}}t)\right]. \quad (23)$$

Transforming above equation (22) into normal mode coordinates x_ω-o-y_ω using coordinate-transformation matrix $\mathbf{p}$ leads to

$$\ddot{x}_\omega + \gamma_{\mathrm{II}}\dot{x}_\omega + (\omega_{\text{II-1}}^2 + \Delta_{\mathrm{p}}\sin^2\theta)x_\omega + \Delta_{\mathrm{p}}\cos\theta\sin\theta y_\omega = F_1\cos(\omega_{\mathrm{d}}t), \quad (24)$$

$$\ddot{y}_\omega + \gamma_{\mathrm{II}}\dot{y}_\omega + (\omega_{\text{II-2}}^2 + \Delta_{\mathrm{p}}\cos^2\theta)y_\omega + \Delta_{\mathrm{p}}\cos\theta\sin\theta x_\omega = F_2\cos(\omega_{\mathrm{d}}t), \quad (25)$$

where

$$F_1 = \frac{2A_{\mathrm{d}}\epsilon_0 V_{\mathrm{d}}V_0}{d_0^2 m_{\mathrm{II}}}\cos\theta, \quad (26)$$

$$F_2 = \frac{2A_{\mathrm{d}}\epsilon_0 V_{\mathrm{d}}V_0}{d_0^2 m_{\mathrm{II}}}\sin\theta. \quad (27)$$

A_{d} and A_{p} are the area of drive and pump electrodes, respectively.

2. *Dynamical sideband coupling simulation based on rotating-frame approximation*

Suppose the solutions of equations (24,25) can be written as [3]

$$x_\omega = \sum_{m=-\infty}^{+\infty}\left[A_m(t)e^{i(\omega_{\mathrm{d}}+m\omega_{\mathrm{p}})t} + cc\right], \quad (28)$$

$$y_\omega = \sum_{m=-\infty}^{+\infty}\left[B_m(t)e^{i(\omega_{\mathrm{d}}+m\omega_{\mathrm{p}})t} + cc\right], \quad (29)$$

where A_m and B_m are slowly varying complex amplitudes of the m-th order idler resonance, and m is any integer. Substituting them into equations (24,25), we obtain

$$\begin{aligned}&\left[2i(\omega_{\mathrm{d}} + m\omega_{\mathrm{p}}) + \gamma_{\mathrm{II}}\right]\dot{A}_m + \left[-(\omega_{\mathrm{d}} + m\omega_{\mathrm{p}})^2 + i\gamma_{\mathrm{II}}(\omega_{\mathrm{d}} + m\omega_{\mathrm{p}}) + \omega_{\text{II-1}}^2 - \frac{\kappa V_{\mathrm{p}}^2}{2}\sin^2\theta\right]A_m \\ &+ \kappa V_0V_{\mathrm{p}}\sin^2\theta\,[A_{m-1} + A_{m+1}] - \frac{\kappa V_{\mathrm{p}}^2}{4}\sin^2\theta\,[A_{m-2} + A_{m+2}] - \frac{\kappa V_{\mathrm{p}}^2}{2}\cos\theta\sin\theta B_m \\ &+ \kappa V_0V_{\mathrm{p}}\cos\theta\sin\theta\,[B_{m-1} + B_{m+1}] - \frac{\kappa V_{\mathrm{p}}^2}{4}\cos\theta\sin\theta\,[B_{m-2} + B_{m+2}] = \frac{F_1}{2}\delta_{m,0}, \quad (30)\\ &\left[2i(\omega_{\mathrm{d}} + m\omega_{\mathrm{p}}) + \gamma_{\mathrm{II}}\right]\dot{B}_m + \left[-(\omega_{\mathrm{d}} + m\omega_{\mathrm{p}})^2 + i\gamma_{\mathrm{II}}(\omega_{\mathrm{d}} + m\omega_{\mathrm{p}}) + \omega_{\text{II-2}}^2 - \frac{\kappa V_{\mathrm{p}}^2}{2}\cos^2\theta\right]B_m \\ &+ \kappa V_0V_{\mathrm{p}}\cos^2\theta\,[B_{m-1} + B_{m+1}] - \frac{\kappa V_{\mathrm{p}}^2}{4}\cos^2\theta\,[B_{m-2} + B_{m+2}] - \frac{\kappa V_{\mathrm{p}}^2}{2}\cos\theta\sin\theta A_m \\ &+ \kappa V_0V_{\mathrm{p}}\cos\theta\sin\theta\,[A_{m-1} + A_{m+1}] - \frac{\kappa V_{\mathrm{p}}^2}{4}\cos\theta\sin\theta\,[A_{m-2} + A_{m+2}] = \frac{F_2}{2}\delta_{m,0}. \quad (31)\end{aligned}$$

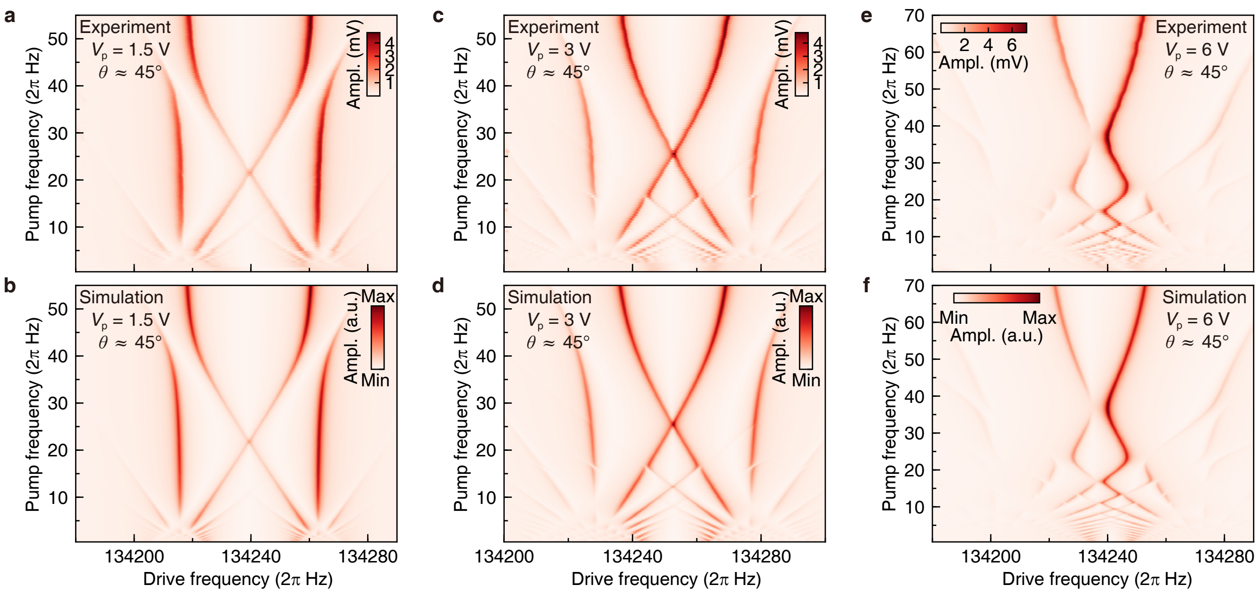

Supplementary Figure 3. The drive frequency ω_{d} and pump frequency ω_{p} responses along x axis detected at ω_{d} when $\theta \approx 45°$ and $\Delta\omega \approx 2\pi \times 40.7$ Hz. **a, b** Experimental (**a**) and simulation (**b**) results with $V_{\mathrm{p}} = 1.5$ V. **c, d** Experimental (**c**) and simulation (**d**) results with $V_{\mathrm{p}} = 3$ V. **e-f** Experimental (**e**) and simulation (**f**) results with $V_{\mathrm{p}} = 6$ V. Source data are provided as a Source Data file.

In above equations, $\delta_{m,0}$ denotes the Kronecker delta function. The second time derivatives of the complex amplitudes have been neglected. For finite order (M) of approximation, the frequency responses of normal modes at ω_{d} can be obtained by applying steady-state condition $\dot{A}_m = \dot{B}_m = 0$ and calculating $|A_0|$ and $|B_0|$, and neglecting the higher-order ($m > M$) idlers. The experimental frequency responses can be reproduced by transforming amplitudes of $x_\omega - o - y_\omega$ to $x - o - y$ using coordinate-transformation matrix $\mathbf{p}$. The ninth-order simulation ($M = 9$) based on this method when θ is tuned to be 36° is shown in Figure 2g of the main file. The simulations of drive and pump frequency responses when $\theta \approx 45°$ and $V_{\mathrm{p}} = 1.5$ V, 3 V, or 6 V are shown in Figure **3**b, d, f, respectively. The corresponding experimental results are shown in Figure **3**a, c, e. The V_{p} dependence of the first-order mode splitting can be simulated by setting $\omega_{\mathrm{p}} = \omega_{\text{II-2}} - \omega_{\text{II-1}}$ and varying V_{p}.

Here we consider the 1-order harmonic pump. the DC and 2-order harmonic terms in the pump are neglected. The dynamics are governed by

$$\ddot{x}_\omega + \gamma_{\mathrm{II}}\dot{x}_\omega + \left[\omega_{\text{II-1}}^2 + \Gamma_1 \cos(\omega_{\mathrm{p}} t)\right] x_\omega + \Lambda \cos(\omega_{\mathrm{p}} t) y_\omega = F_1 \cos(\omega_{\mathrm{d}} t), \tag{32}$$

$$\ddot{y}_\omega + \gamma_{\mathrm{II}}\dot{y}_\omega + \left[\omega_{\text{II-2}}^2 + \Gamma_2 \cos(\omega_{\mathrm{p}} t)\right] y_\omega + \Lambda \cos(\omega_{\mathrm{p}} t) x_\omega = F_2 \cos(\omega_{\mathrm{d}} t), \tag{33}$$

where Γ_i $(i = 1, 2)$ and Λ are the coefficients of intra- and inter-modal coupling, respectively. $\Gamma_1 = 2\kappa V_0 V_{\mathrm{p}} \sin^2\theta$, $\Gamma_2 = 2\kappa V_0 V_{\mathrm{p}} \cos^2\theta$, $\Lambda = 2\kappa V_0 V_{\mathrm{p}} \cos\theta \sin\theta$. The multiple-scale analysis [4] is used to interpretation the coupling process. First, we introduce dimensionless variables $t = \omega_0 t^*$, $u = x_\omega/d_0$, and $v = y_\omega/d_0$, where t^* denotes the real time, $\omega_0^2 = \left(\omega_{\text{II-1}}^2 + \omega_{\text{II-2}}^2\right)/2$, d_0 is the initial capacitive gap. By then introducing small parameter $\epsilon = \gamma_{\mathrm{II}}/\omega_0$, equations (32,33) can be nondimensionalized as

$$D^2 u + \epsilon D u + \left[\omega_1^2 + \epsilon\mu_1 \cos(\Omega_{\mathrm{p}} t)\right] u + \epsilon\lambda \cos(\Omega_{\mathrm{p}} t) v = \epsilon f_1 \cos(\Omega_{\mathrm{d}} t), \tag{34}$$

$$D^2 v + \epsilon D v + \left[\omega_2^2 + \epsilon\mu_2 \cos(\Omega_{\mathrm{p}} t)\right] v + \epsilon\lambda \cos(\Omega_{\mathrm{p}} t) u = \epsilon f_2 \cos(\Omega_{\mathrm{d}} t), \tag{35}$$

where $D = d/dt$, $D^2 = d^2/dt^2$, $\omega_i = \omega_{\text{II-i}}/\omega_0$, $\Omega_{\mathrm{p}} = \omega_{\mathrm{p}}/\omega_0$, $\Omega_{\mathrm{d}} = \omega_{\mathrm{d}}/\omega_0$, $\mu_i = \Gamma_i/(\omega_0\gamma_{\mathrm{II}})$, $\lambda = \Lambda/(\omega_0\gamma_{\mathrm{II}})$, $f_i = F_i/(\omega_0 d_0 \gamma_{\mathrm{II}})$, $(i = 1, 2)$.

We define multiple times scales $T_0 = t, T_1 = \epsilon t, T_2 = \epsilon^2 t, \cdots$ The solutions can be expressed as

$$u = u_0(T_0, T_1, T_2) + \epsilon u_1(T_0, T_1, T_2) + \epsilon^2 u_2(T_0, T_1, T_2), \tag{36}$$

$$v = v_0(T_0, T_1, T_2) + \epsilon v_1(T_0, T_1, T_2) + \epsilon^2 v_2(T_0, T_1, T_2). \tag{37}$$

Using chain rule, we have $D = D_0 + \epsilon D_1$, and $D^2 = D_0^2 + 2\epsilon D_0 D_1 + \epsilon^2(2D_0 D_2 + D_1^2)$. The higher order terms are neglected. As described previously, D_m^n denotes the n-th order differential operator with respect to T_m, $(m = 0, 1, 2.\ n = 1, 2)$. Substituting (36,37) into (34,35), and equating the coefficients of like power of ϵ, we obtain
Order ϵ^0

$$D_0^2 u_0 + \omega_1^2 u_0 = 0, \tag{38}$$

$$D_0^2 v_0 + \omega_2^2 v_0 = 0. \tag{39}$$

Order ϵ^1

$$D_0^2 u_1 + \omega_1^2 u_1 = -2D_0 D_1 u_0 - D_0 u_0 - \mu_1 u_0 \cos(\Omega_\mathrm{p} T_0) - \lambda v_0 \cos(\Omega_\mathrm{p} T_0) + f_1 \cos(\Omega_\mathrm{d} T_0), \tag{40}$$

$$D_0^2 v_1 + \omega_2^2 v_1 = -2D_0 D_1 v_0 - D_0 v_0 - \mu_2 v_0 \cos(\Omega_\mathrm{p} T_0) - \lambda u_0 \cos(\Omega_\mathrm{p} T_0) + f_2 \cos(\Omega_\mathrm{d} T_0). \tag{41}$$

Order ϵ^2

$$\begin{aligned} D_0^2 u_2 + \omega_1^2 u_2 &= -2D_0 D_2 u_0 - D_1^2 u_0 - D_1 u_0 - 2D_0 D_1 u_1 - D_0 u_1 - \mu_1 u_1 \cos(\Omega_\mathrm{p} T_0) \\ &- \lambda v_1 \cos(\Omega_\mathrm{p} T_0), \end{aligned} \tag{42}$$

$$\begin{aligned} D_0^2 v_2 + \omega_2^2 v_2 &= -2D_0 D_2 v_0 - D_1^2 v_0 - D_1 v_0 - 2D_0 D_1 v_1 - D_0 v_1 - \mu_2 v_1 \cos(\Omega_\mathrm{p} T_0) \\ &- \lambda u_1 \cos(\Omega_\mathrm{p} T_0). \end{aligned} \tag{43}$$

The general solutions of (38) and (39) can be written as

$$u_0 = A(T_1, T_2) \exp(i\omega_1 T_0) + \bar{A}(T_1, T_2) \exp(-i\omega_1 T_0), \tag{44}$$

$$v_0 = B(T_1, T_2) \exp(i\omega_2 T_0) + \bar{B}(T_1, T_2) \exp(-i\omega_2 T_0). \tag{45}$$

Substituting (44) and (45) into (40) and (41) leads to

$$\begin{aligned} D_0^2 u_1 + \omega_1^2 u_1 &= \underbrace{(-2i\omega_1 D_1 A - i\omega_1 A) \exp(i\omega_1 T_0)}_{\text{Homogeneous secular term}} - \frac{\mu_1 A}{2} \exp[i(\omega_1 + \Omega_\mathrm{p}) T_0] \\ &- \frac{\mu_1 A}{2} \exp[i(\omega_1 - \Omega_\mathrm{p}) T_0] - \frac{\lambda B}{2} \exp[i(\omega_2 + \Omega_\mathrm{p}) T_0] - \frac{\lambda B}{2} \exp[i(\omega_2 - \Omega_\mathrm{p}) T_0] \\ &+ \frac{f_1}{2} \exp(i\Omega_\mathrm{d} T_0) + cc, \end{aligned} \tag{46}$$

$$\begin{aligned} D_0^2 v_1 + \omega_2^2 v_1 &= \underbrace{(-2i\omega_2 D_1 B - i\omega_2 B) \exp(i\omega_2 T_0)}_{\text{Homogeneous secular term}} - \frac{\mu_2 B}{2} \exp[i(\omega_2 + \Omega_\mathrm{p}) T_0] \\ &- \frac{\mu_2 B}{2} \exp[i(\omega_2 - \Omega_\mathrm{p}) T_0] - \frac{\lambda A}{2} \exp[i(\omega_1 + \Omega_\mathrm{p}) T_0] - \frac{\lambda A}{2} \exp[i(\omega_1 - \Omega_\mathrm{p}) T_0] \\ &+ \frac{f_2}{2} \exp(i\Omega_\mathrm{d} T_0) + cc. \end{aligned} \tag{47}$$

For the first-order dynamical coupling case, $\Omega_\mathrm{p} \approx \omega_2 - \omega_1$. We introduce pump detuning parameter σ_p, which is defined by $\Omega_\mathrm{p} = \omega_2 - \omega_1 + \epsilon\sigma_\mathrm{p}$. The $\omega_j \pm \Omega_\mathrm{p}$ ($j = 1, 2$) tones (sidebands) are generated by wave mixing in (46) and (47). Apart from the homogeneous secular terms, the inter-modal coupling term will cause inhomogeneous secular terms $-\lambda B/2 \exp(-i\sigma_\mathrm{p} T_1)$ in (46) and

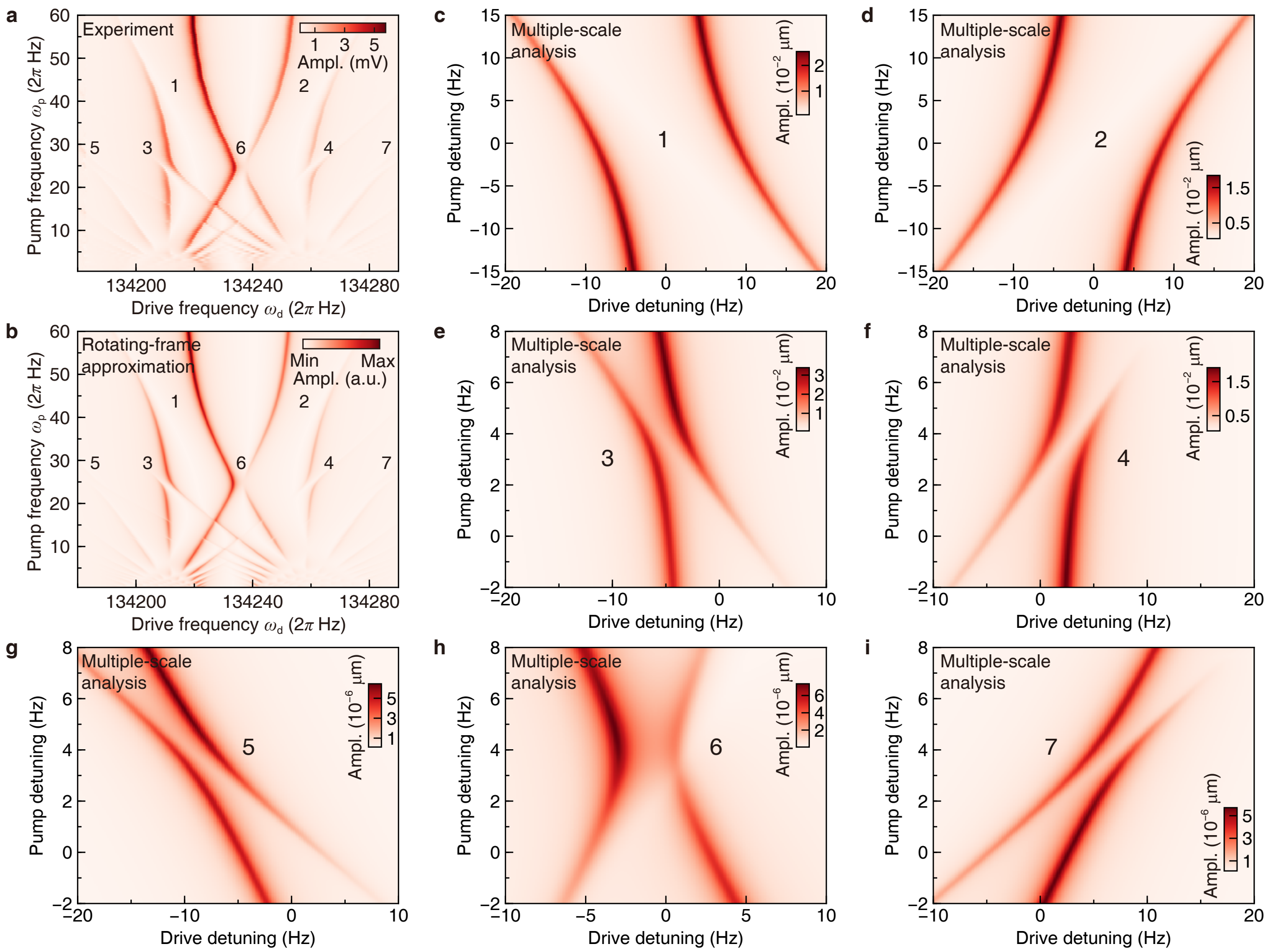

Supplementary Figure 4. Interpretation of the dynamical coupling based on multiple-scale analysis. **a** Experimental results of the dynamical coupling (identical to Figure 3c of the paper). **b** Simulation based on rotating-frame approximation (identical to Figure 3d of the paper). **c-i** Simulation of the avoided crossings based on multiple-scale analysis when (**c**) $\omega_d \approx \omega_{\text{II-1}}$ and $\omega_p \approx \omega_{\text{II-2}} - \omega_{\text{II-1}}$, (**d**) $\omega_d \approx \omega_{\text{II-2}}$ and $\omega_p \approx \omega_{\text{II-2}} - \omega_{\text{II-1}}$, (**e**) $\omega_d \approx \omega_{\text{II-1}}$ and $\omega_p \approx (\omega_{\text{II-2}} - \omega_{\text{II-1}})/2$, (**f**) $\omega_d \approx \omega_{\text{II-2}}$ and $\omega_p \approx (\omega_{\text{II-2}} - \omega_{\text{II-1}})/2$, (**g**) $\omega_d \approx (3\omega_{\text{II-1}} - \omega_{\text{II-2}})/2$ and $\omega_p \approx (\omega_{\text{II-2}} - \omega_{\text{II-1}})/2$, (**h**) $\omega_d \approx (\omega_{\text{II-1}} + \omega_{\text{II-2}})/2$ and $\omega_p \approx (\omega_{\text{II-2}} - \omega_{\text{II-1}})/2$, (**i**) $\omega_d \approx (3\omega_{\text{II-2}} - \omega_{\text{II-1}})/2$ and $\omega_p \approx (\omega_{\text{II-2}} - \omega_{\text{II-1}})/2$. Source data are provided as a Source Data file.

$-\lambda A/2 \exp(i\sigma_p T_1)$ in (47), indicating that sidebands 2 and 3 in Figure 2d of the main file will generate resonances. Of course, external forces will also induce inhomogeneous secular terms if $\Omega_d \approx \omega_1$ or ω_2. Here, we also introduce drive detuning parameter σ_d that is defined by $\Omega_d = \omega_i + \epsilon\sigma_d$, ($i = 1$ or 2). The inter-modal coupling induced secular terms will actuate one mode using another mode's displacement. When the system is externally excited, the coupling term will cause energy exchange between the two modes. Suppose the drive force is actuated at mode II-1, which means

$\Omega_{\mathrm{p}} \approx \omega_2 - \omega_1$ and $\Omega_{\mathrm{d}} \approx \omega_1$. Annihilating the secular terms leads to

$$-2i\omega_1 D_1 A - i\omega_1 A \underbrace{-\frac{\lambda B}{2}\exp\left(-i\sigma_{\mathrm{p}} T_1\right)}_{\text{Sideband 2}} + \frac{f_1}{2}\exp\left(i\sigma_{\mathrm{d}} T_1\right) = 0, \tag{48}$$

$$-2i\omega_2 D_1 B - i\omega_2 B \underbrace{-\frac{\lambda A}{2}\exp\left(i\sigma_{\mathrm{p}} T_1\right)}_{\text{Sideband 3}} = 0. \tag{49}$$

To solve those equations, we write the complex amplitudes into polar form $A = |u|\exp\left(i\phi_1\right)/2$ and $B = |v|\exp\left(i\phi_2\right)/2$, where $|u|$ and $|v|$ are the first approximations of the steady-state amplitudes of modes II-1 and II-2, and ϕ_1 and ϕ_2 are the first approximations of their phases. Substituting them into (48,49) we obtain

$$|u| = \frac{f_1}{\sqrt{\left[2\omega_1\sigma_{\mathrm{d}} - \frac{(\sigma_{\mathrm{d}}+\sigma_{\mathrm{p}})\lambda^2}{8\omega_2(\sigma_{\mathrm{d}}+\sigma_{\mathrm{p}})^2+2\omega_2}\right]^2 + \left[\omega_1 + \frac{\lambda^2}{16\omega_2(\sigma_{\mathrm{d}}+\sigma_{\mathrm{p}})^2+4\omega_2}\right]^2}}, \tag{50}$$

$$|v| = \frac{\lambda}{2\omega_2\sqrt{4(\sigma_{\mathrm{d}}+\sigma_{\mathrm{p}})^2+1}}|u|. \tag{51}$$

The experimentally observed avoided crossing can be reproduced by transforming normal modes amplitudes into hybrid modes amplitudes using coordinate-transformation matrix **p** (Fig.**4**c). The steady-state frequency response of the first-order dynamical coupling when $\Omega_{\mathrm{p}} \approx \omega_2 - \omega_1$ and $\Omega_{\mathrm{d}} \approx \omega_2$ can also be obtained similarly (Fig.**4**d).

In order to demonstrate the interaction between the external force and sidebands, we assume $\sigma_{\mathrm{d}} = \sigma_{\mathrm{p}} = 0$ and apply the steady-state condition $D_1 A = D_1 B = 0$. Solving equations (48,49), we obtain $B = f_1/(\frac{4\omega_1\omega_2}{\lambda} + \lambda)$ and $A = -2i\omega_2 f_1/(4\omega_1\omega_2 + \lambda^2)$, which indicate that displacement of mode II-2 is in phase with external force, while displacement of mode II-1 is in quadrature with external force. Sideband 2 in (48) is in antiphase with external force. In other words, sideband 2 is destructive to the external force for red-detuned pump condition, thus will cause cooling or avoided crossing (mode splitting).

To calculate the mode splitting, we make $\sigma_{\mathrm{p}} = 0$ in (50). The coupling rate g_1 can be obtained by finding the value of σ_{d} that makes the denominator of $|u|$ the minimum, which is given by $\sigma_{\mathrm{d}0} = \sqrt{\lambda^2/16\omega_1\omega_2 - 1/4}$. g_1 is given by

$$g_1 = 2\epsilon\omega_0\sigma_{\mathrm{d}0} = \sqrt{\frac{\Lambda^2}{4\omega_{\text{II-1}}\omega_{\text{II-2}}} - \gamma_{\mathrm{II}}^2}. \tag{52}$$

For the second-order dynamical coupling case, $\Omega_{\mathrm{p}} \approx (\omega_2 - \omega_1)/2$. The pump will not produce secular term in (46) and (47). Thus, we turn to higher order equations (42) and (43). Suppose the

external force is driving at II-1 mode, $\Omega_{\mathrm{d}} = \omega_1 + \epsilon\sigma_{\mathrm{d}}$ (The analysis of driving at II-2 mode are similar). The particular solutions of (46) and (47) after eliminating the secular terms are

$$u_1 = \frac{\mu_1 A}{2}\frac{\exp\left[i(\omega_1+\Omega_{\mathrm{p}})T_0\right]}{\Omega_{\mathrm{p}}^2+2\omega_1\Omega_{\mathrm{p}}} + \frac{\mu_1 A}{2}\frac{\exp\left[i(\omega_1-\Omega_{\mathrm{p}})T_0\right]}{\Omega_{\mathrm{p}}^2-2\omega_1\Omega_{\mathrm{p}}} + \frac{\lambda B}{2}\frac{\exp\left[i(\omega_2+\Omega_{\mathrm{p}})T_0\right]}{(\omega_2+\Omega_{\mathrm{p}})^2-\omega_1^2}$$
$$+ \frac{\lambda B}{2}\frac{\exp\left[i(\omega_2-\Omega_{\mathrm{p}})T_0\right]}{(\omega_2-\Omega_{\mathrm{p}})^2-\omega_1^2} + cc, \tag{53}$$
$$v_1 = \frac{\mu_2 B}{2}\frac{\exp\left[i(\omega_2+\Omega_{\mathrm{p}})T_0\right]}{\Omega_{\mathrm{p}}^2+2\omega_2\Omega_{\mathrm{p}}} + \frac{\mu_2 B}{2}\frac{\exp\left[i(\omega_2-\Omega_{\mathrm{p}})T_0\right]}{\Omega_{\mathrm{p}}^2-2\omega_2\Omega_{\mathrm{p}}} + \frac{\lambda A}{2}\frac{\exp\left[i(\omega_1+\Omega_{\mathrm{p}})T_0\right]}{(\omega_1+\Omega_{\mathrm{p}})^2-\omega_2^2}$$
$$+ \frac{\lambda A}{2}\frac{\exp\left[i(\omega_1-\Omega_{\mathrm{p}})T_0\right]}{(\omega_1-\Omega_{\mathrm{p}})^2-\omega_2^2} + \frac{f_2}{2}\frac{\exp\left(i\Omega_{\mathrm{d}}T_0\right)}{\omega_2^2-\Omega_{\mathrm{d}}^2} + cc. \tag{54}$$

Substituting (44), (45), (53), and (54) into (42) and (43), we can find that wave mixing will produce $\omega_j \pm \Omega_{\mathrm{p}} \pm \Omega_{\mathrm{p}}$ $(j = 1, 2)$ tones (sidebands), which could produce additional secular terms.

$$D_0^2 u_2 + \omega_1^2 u_2 = -\left(2i\omega_1 D_2 A + D_1^2 A + D_1 A\right)\exp\left(i\omega_1 T_0\right) - \frac{\mu_1^2 A}{2}\frac{\exp\left(i\omega_1 T_0\right)}{\Omega_{\mathrm{p}}^2-4\omega_1^2}$$
$$-\frac{\mu_1\lambda B}{4}\frac{\exp\left[i(\omega_2-2\Omega_{\mathrm{p}})T_0\right]}{(\omega_2-\Omega_{\mathrm{p}})^2-\omega_1^2} - \frac{\lambda^2 A}{4}\left[\frac{1}{(\omega_1+\Omega_{\mathrm{p}})^2-\omega_2^2}+\frac{1}{(\omega_1-\Omega_{\mathrm{p}})^2-\omega_2^2}\right]\exp\left(i\omega_1 T_0\right)$$
$$-\frac{\mu_2\lambda B}{4}\frac{\exp\left[i(\omega_2-2\Omega_{\mathrm{p}})T_0\right]}{\Omega_{\mathrm{p}}^2-2\omega_2\Omega_{\mathrm{p}}} + cc + \infty, \tag{55}$$
$$D_0^2 v_2 + \omega_2^2 v_2 = -\left(2i\omega_2 D_2 B + D_1^2 B + D_1 B\right)\exp\left(i\omega_2 T_0\right) - \frac{\mu_2^2 B}{2}\frac{\exp\left(i\omega_2 T_0\right)}{\Omega_{\mathrm{p}}^2-4\omega_2^2}$$
$$-\frac{\mu_2\lambda A}{4}\frac{\exp\left[i(\omega_1+2\Omega_{\mathrm{p}})T_0\right]}{(\omega_1+\Omega_{\mathrm{p}})^2-\omega_2^2} - \frac{\lambda^2 B}{4}\left[\frac{1}{(\omega_2+\Omega_{\mathrm{p}})^2-\omega_1^2}+\frac{1}{(\omega_2-\Omega_{\mathrm{p}})^2-\omega_1^2}\right]\exp\left(i\omega_2 T_0\right)$$
$$-\frac{\mu_1\lambda A}{4}\frac{\exp\left[i(\omega_1+2\Omega_{\mathrm{p}})T_0\right]}{\Omega_{\mathrm{p}}^2+2\omega_1\Omega_{\mathrm{p}}} + cc + \infty, \tag{56}$$

where ∞ denotes the non-secular terms. The steady-state frequency responses of the second-order dynamical coupling can be obtained by eliminating all the secular terms in (46), (47), (55), and (56) (Fig.**4**e). The second-order coupling strength g_2 can be obtained by finding the value of σ_{d} that makes the frequency-response amplitude the maximum, which is given by

$$\sigma'_{\mathrm{d0}} \approx \frac{\epsilon\lambda\left(\frac{\mu_2}{2\omega_1+3\Omega_{\mathrm{p}}}-\frac{\mu_1}{2\omega_1+\Omega_{\mathrm{p}}}\right)}{8\Omega_{\mathrm{p}}\sqrt{\omega_1\omega_2}}. \tag{57}$$

Then, g_2 is obtained,

$$g_2 = 2\epsilon\omega_0\sigma'_{\mathrm{d0}} \approx \frac{\Lambda\left(\frac{\Gamma_2}{\omega_{\text{II-1}}+3\omega_{\text{II-2}}}-\frac{\Gamma_1}{3\omega_{\text{II-1}}+\omega_{\text{II-2}}}\right)}{2(\omega_{\text{II-2}}-\omega_{\text{II-1}})\sqrt{\omega_{\text{II-1}}\omega_{\text{II-2}}}}$$
$$\approx \frac{2\kappa^2 V_0^2 V_{\mathrm{p}}^2\sin 2\theta\left(\frac{\cos^2\theta}{\omega_{\text{II-1}}+3\omega_{\text{II-2}}}-\frac{\sin^2\theta}{3\omega_{\text{II-1}}+\omega_{\text{II-2}}}\right)}{2(\omega_{\text{II-2}}-\omega_{\text{II-1}})\sqrt{\omega_{\text{II-1}}\omega_{\text{II-2}}}}. \tag{58}$$

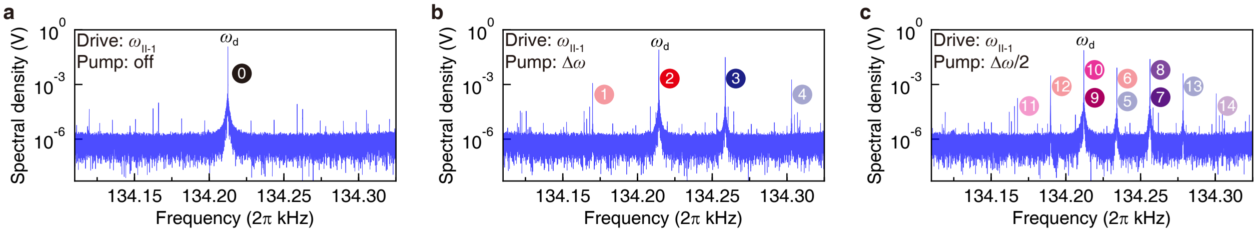

Supplementary Figure 5. **a-c** Spectrum diagram tested along x axis when (**a**) drive at mode II-1 without pump, (**b**) drive at mode II-1 with pump frequency $\omega_\mathrm{p} \approx \Delta\omega$, and (**c**) drive at mode II-1 with pump frequency $\omega_\mathrm{p} \approx \Delta\omega/2$. The sideband numbers in each spectrum line are depicted. Source data are provided as a Source Data file.

Likewise, the second-order coupling when external force is driving at II-2 mode ($\Omega_\mathrm{d} \approx \omega_2$) can also be simulated (Fig.**4**f). When the forces are not driving at resonance condition, but driving at a frequency that is half the frequency difference away from one of the resonance frequencies, they will also provide inhomogeneous secular terms in (55) and (56). Combined with mode coupling induced secular terms, avoided crossing could take place at those frequencies (Fig.**4**g-i). Here we only analyse the first- and second-order approximations. For higher-order approximations, the $\omega_j \pm \Omega_\mathrm{p} \pm \Omega_\mathrm{p} \pm \Omega_\mathrm{p} \pm \cdots$ ($j = 1, 2$) tones will be generated, which will provide higher-order couplings.

The sideband processes of the first- and second-order dynamical coupling can be revealed by the spectrum graphs. When mode II-1 is actuated without pump, the spectrum line 0 reveals the drive tone (Fig.**5**a). When drive at mode II-1 with pump frequency $\omega_\mathrm{p} \approx \Delta\omega$, the sideband driven spectrum lines are shown in Figure **5**b. When drive at mode II-1 with pump frequency $\omega_\mathrm{p} \approx \Delta\omega/2$, the sideband driven spectrum lines are shown in Figure **5**c.

4. *Effect of the DC term in the pump*

As depicted in equation (23), there is a DC term $-\kappa\frac{V_\mathrm{p}^2}{2}$ in the pump, which is applied along y direction. Based on the electrostatic tuning theory in Section Supplementary Note 2 of the Supplementary Information, we can see that this DC term will tune the hybrid coupling condition, thus affect resonant frequencies and misalignment angle θ.

Repeating the sweeps depicted in Figure 3f by applying non-resonance pumps with different values of V_p. In this condition only the DC term in the pump signal would affect the system. The effect of DC term on the order-2 modes is obtained, as shown in Figure **6**. The dashed lines are theoretical values obtained using the electrostatic tuning theory.

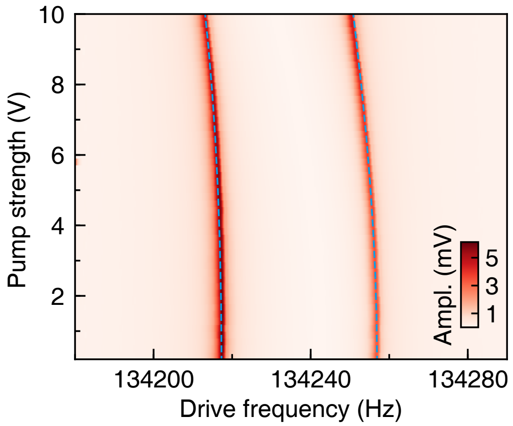

Supplementary Figure 6. The effect of the DC term in the pump on the order-2 modes. Source data are provided as a Source Data file.

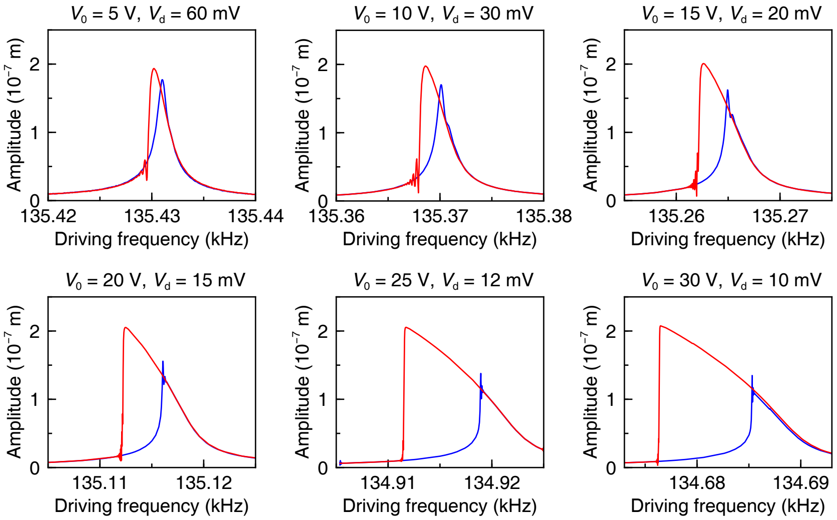

Supplementary Figure 7. Frequency responses of order-2 mode with different drive conditions. V_0 is the DC voltage applied on the resonator body, and V_d is the amplitude of the AC drive voltage applied on drive electrodes. V_0 is changed while $V_0 \times V_\mathrm{d}$ is kept constant in these tests. Red (blue) curves are responses that are swept downward (upward). Source data are provided as a Source Data file.

Supplementary Note 4. Electrostatic nonlinear coupling of modes II and III

1. Dominance of the electrostatic nonlinearity

The ring resonator in this resonator is dominated by stiffness-softening electrostatic nonlinearity, which is confirmed by applying different values of DC voltage V_0 while keep the displacement amplitude almost constant (Fig. **7**, Fig. **8**). The constant displacement amplitude can be approximately

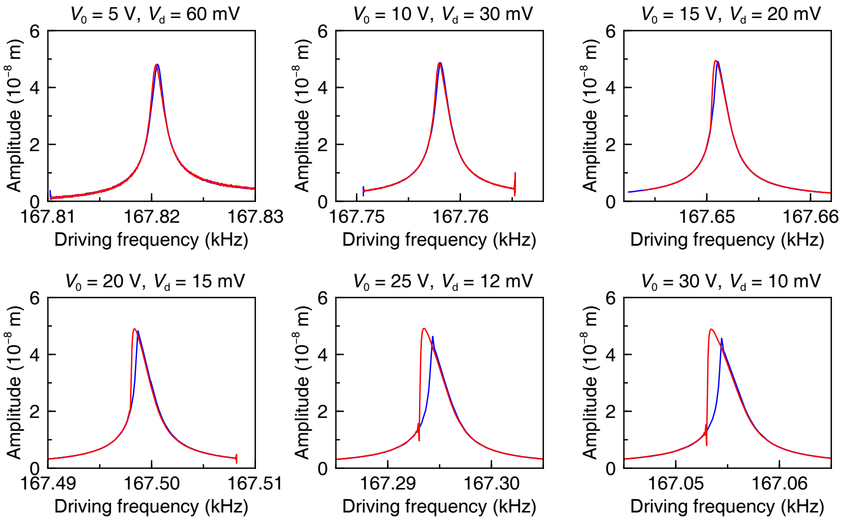

Supplementary Figure 8. Frequency responses of order-3 mode with different drive conditions. V_0 is the DC voltage applied on the resonator body, and V_d is the amplitude of the AC drive voltage applied on drive electrodes. V_0 is changed while $V_0 \times V_\mathrm{d}$ is kept constant in these tests. Red (blue) curves are responses that are swept downward (upward). Source data are provided as a Source Data file.

achieved by applying constant drive force, because V_0 induced frequency shift is negligible compared to the eigenfrequencies. We change the value of the DC voltage V_0, while keep the product of the DC voltage and AC drive voltage amplitude $V_0 \times V_\mathrm{d}$ (thus the drive force equation (5)) unchanged, and implement the frequency-response tests. The ring resonator in this experiment was co-fabricated with the device characterized in other experiments of this paper. They have the same structural design and doping condition. The results for order-2 and order-3 modes are shown in Fig. **7** and Fig. **8**, respectively. This resonator shows stiffness-softening Duffing nonlinear frequency responses, and the nonlinearity strength is highly DC voltage-dependent. The electrostatic nonlinearity is sensitive to the DC voltage whereas other nonlinearities are not. Based on the results depicted in Figure **7** and Figure **8**), we can draw a conclusion that the ring resonator used in this paper is dominated by electrostatic nonlinearity when V_0 is set to be 30 V.

2. *Electrostatic parametric coupling*

The coupling is described by two mechanical modes sharing a biased capacitor. When one resonator oscillates, the capacitance is alternatively changed, which will also alternatively modifying the other modes effective stiffness. The equations of motion can be obtained using Lagrangian method.

The potential energy U and the kinetic energy T of the system are given by

$$U = \frac{k_{\mathrm{II}} X_{\mathrm{II}}^2}{2} + \frac{k_{\mathrm{III}} X_{\mathrm{III}}^2}{2} - \frac{A_{\mathrm{c}} \epsilon_0 \Delta V^2}{2(d_0 + X_{\mathrm{II}} + X_{\mathrm{III}})}, \tag{59}$$

$$T = \frac{m_{\mathrm{II}} \dot{X}_{\mathrm{II}}^2}{2} + \frac{m_{\mathrm{III}} \dot{X}_{\mathrm{III}}^2}{2}, \tag{60}$$

where $k_\square$, $m_\square$, and $X_\square$ ($\square = \mathrm{II}, \mathrm{III}$) are the stiffness, mass and the displacement respect to initial equilibrium position, respectively. ΔV is the bias voltage applied on the capacitor. A_{c} and d_0 are the area and initial gap of the capacitor, respectively. Substituting them into Lagrangian function,

$$\frac{\mathrm{d}}{\mathrm{d}t} \frac{\partial T}{\partial \dot{X}_\square} - \frac{\partial T}{\partial X_\square} + \frac{\partial U}{\partial X_\square} = 0, (\square = \mathrm{II}, \mathrm{III}). \tag{61}$$

The equations of motion can be obtained

$$m_{\mathrm{II}} \ddot{X}_{\mathrm{II}} + k_{\mathrm{II}} X_{\mathrm{II}} + \frac{A_{\mathrm{c}} \epsilon_0 \Delta V^2}{2(d_0 + X_{\mathrm{II}} + X_{\mathrm{III}})^2} = 0, \tag{62}$$

$$m_{\mathrm{III}} \ddot{X}_{\mathrm{III}} + k_{\mathrm{III}} X_{\mathrm{III}} + \frac{A_{\mathrm{c}} \epsilon_0 \Delta V^2}{2(d_0 + X_{\mathrm{II}} + X_{\mathrm{III}})^2} = 0. \tag{63}$$

Expanding the nonlinear restoring force in to Taylor series respect to X_{II} and X_{III} leads to

$$\begin{aligned} & m_{\mathrm{II}} \ddot{x}_{\mathrm{II}} + k_{\mathrm{II}} x_{\mathrm{II}} + k_{\mathrm{II}} X_{\mathrm{II},0} + \frac{A_{\mathrm{c}} \epsilon_0 \Delta V^2}{2(d_0 + X_{\mathrm{II},0} + X_{\mathrm{III},0})^2} - \frac{A_{\mathrm{c}} \epsilon_0 \Delta V^2}{(d_0 + X_{\mathrm{II},0} + X_{\mathrm{III},0})^3} (x_{\mathrm{II}} + x_{\mathrm{III}}) \\ & + \frac{3 A_{\mathrm{c}} \epsilon_0 \Delta V^2}{2(d_0 + X_{\mathrm{II},0} + X_{\mathrm{III},0})^4} (x_{\mathrm{II}} + x_{\mathrm{III}})^2 - \frac{2 A_{\mathrm{c}} \epsilon_0 \Delta V^2}{(d_0 + X_{\mathrm{II},0} + X_{\mathrm{III},0})^5} (x_{\mathrm{II}} + x_{\mathrm{III}})^3 = 0, \end{aligned} \tag{64}$$

$$\begin{aligned} & m_{\mathrm{III}} \ddot{x}_{\mathrm{III}} + k_{\mathrm{III}} x_{\mathrm{III}} + k_{\mathrm{III}} X_{\mathrm{III},0} + \frac{A_{\mathrm{c}} \epsilon_0 \Delta V^2}{2(d_0 + X_{\mathrm{II},0} + X_{\mathrm{III},0})^2} - \frac{A_{\mathrm{c}} \epsilon_0 \Delta V^2}{(d_0 + X_{\mathrm{II},0} + X_{\mathrm{III},0})^3} (x_{\mathrm{II}} + x_{\mathrm{III}}) \\ & + \frac{3 A_{\mathrm{c}} \epsilon_0 \Delta V^2}{2(d_0 + X_{\mathrm{II},0} + X_{\mathrm{III},0})^4} (x_{\mathrm{II}} + x_{\mathrm{III}})^2 - \frac{2 A_{\mathrm{c}} \epsilon_0 \Delta V^2}{(d_0 + X_{\mathrm{II},0} + X_{\mathrm{III},0})^5} (x_{\mathrm{II}} + x_{\mathrm{III}})^3 = 0, \end{aligned} \tag{65}$$

where $x_\square = X_\square - X_{\square,0}$, ($\square = \mathrm{II}, \mathrm{III}$). If $X_{\mathrm{II},0}$ and $X_{\mathrm{III},0}$ are the new equilibrium positions, which satisfy

$$k_{\mathrm{II}} X_{\mathrm{II},0} + \frac{A_{\mathrm{c}} \epsilon_0 \Delta V^2}{2(d_0 + X_{\mathrm{II},0} + X_{\mathrm{III},0})^2} = k_{\mathrm{III}} X_{\mathrm{III},0} + \frac{A_{\mathrm{c}} \epsilon_0 \Delta V^2}{2(d_0 + X_{\mathrm{II},0} + X_{\mathrm{III},0})^2} = 0, \tag{66}$$

and denote $d_1 = d_0 + X_{\mathrm{II},0} + X_{\mathrm{III},0}$, the EOMs can be simplified as

$$\ddot{x}_{\mathrm{II}} + \gamma_{\mathrm{II}} \dot{x}_{\mathrm{II}} + \omega_{\mathrm{II}}^2 x_{\mathrm{II}} + \alpha_{\mathrm{II}} x_{\mathrm{III}} + \beta_{\mathrm{II}} (x_{\mathrm{II}} + x_{\mathrm{III}})^2 + \nu_{\mathrm{II}} (x_{\mathrm{II}} + x_{\mathrm{III}})^3 = 0, \tag{67}$$

$$\ddot{x}_{\mathrm{III}} + \gamma_{\mathrm{III}} \dot{x}_{\mathrm{III}} + \omega_{\mathrm{III}}^2 x_{\mathrm{III}} + \alpha_{\mathrm{III}} x_{\mathrm{II}} + \beta_{\mathrm{III}} (x_{\mathrm{II}} + x_{\mathrm{III}})^2 + \nu_{\mathrm{III}} (x_{\mathrm{II}} + x_{\mathrm{III}})^3 = 0, \tag{68}$$

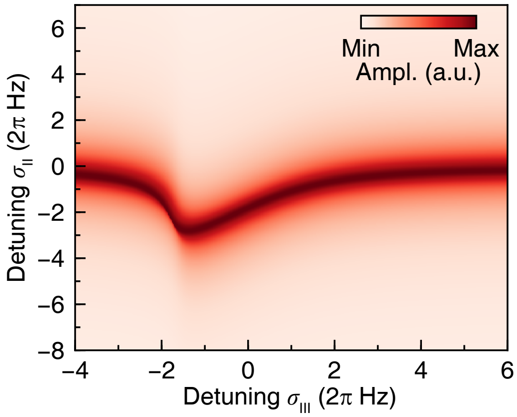

Supplementary Figure 9. Simulation of the dispersive frequency shift of mode II caused by simultaneously actuated mode III based on multiple-scale analysis. Source data are provided as a Source Data file.

where damping rate $\gamma_\square$ is additionally introduced, and

$$\omega_\square^2 = \frac{k_\square}{m_\square} - \frac{A_c\epsilon_0\Delta V^2}{m_\square d_1^3}, \tag{69}$$

$$\alpha_\square = -\frac{A_c\epsilon_0\Delta V^2}{m_\square d_1^3}, \tag{70}$$

$$\beta_\square = \frac{3A_c\epsilon_0\Delta V^2}{2m_\square d_1^4}, \tag{71}$$

$$\nu_\square = -\frac{2A_c\epsilon_0\Delta V^2}{m_\square d_1^5}, (\square = \mathrm{II}, \mathrm{III}). \tag{72}$$

If both mode II and III are simultaneously actuated, the dispersive frequency shift can be simulated by solving the following coupling equations with external forces using multiple-scale analysis.

$$\ddot{x}_{\mathrm{II}} + \gamma_{\mathrm{II}}\dot{x}_{\mathrm{II}} + \omega_{\mathrm{II}}^2 x_{\mathrm{II}} + \alpha_{\mathrm{II}} x_{\mathrm{III}} + \beta_{\mathrm{II}}(x_{\mathrm{II}} + x_{\mathrm{III}})^2 + \nu_{\mathrm{II}}(x_{\mathrm{II}} + x_{\mathrm{III}})^3 = F_{\mathrm{II}} \sin(\omega_{\text{d-II}} t), \tag{73}$$

$$\ddot{x}_{\mathrm{III}} + \gamma_{\mathrm{III}}\dot{x}_{\mathrm{III}} + \omega_{\mathrm{III}}^2 x_{\mathrm{III}} + \alpha_{\mathrm{III}} x_{\mathrm{II}} + \beta_{\mathrm{III}}(x_{\mathrm{II}} + x_{\mathrm{III}})^2 + \nu_{\mathrm{III}}(x_{\mathrm{II}} + x_{\mathrm{III}})^3 = F_{\mathrm{III}} \sin(\omega_{\text{d-III}} t), \tag{74}$$

where $F_\square$ and $\omega_{\text{d-}\square}$ ($\square = \mathrm{II}, \mathrm{III}$) are the amplitudes and frequencies of the external forces. The calculation process will be reported elsewhere. Here, we just give the results. The amplitudes of the modes $|x_{\mathrm{II}}|$ and $|x_{\mathrm{III}}|$ are decided by the following equations,

$$\left(\frac{1}{4}M_1|x_{\mathrm{II}}|^3 + \frac{1}{4}N_1|x_{\mathrm{II}}||x_{\mathrm{III}}|^2 + \frac{2\omega_{\mathrm{II}}|x_{\mathrm{II}}|\sigma_{\mathrm{II}}}{\gamma_{\mathrm{II}}}\right)^2 = f_{\mathrm{II}}^2 - \frac{\omega_{\mathrm{II}}^2|x_{\mathrm{II}}|^2}{\gamma_{\mathrm{II}}^2}, \tag{75}$$

$$\left(\frac{1}{4}M_2|x_{\mathrm{III}}|^3 + \frac{1}{4}N_2|x_{\mathrm{II}}|^2|x_{\mathrm{III}}| + \frac{2\omega_{\mathrm{III}}|x_{\mathrm{III}}|\sigma_{\mathrm{III}}}{\gamma_{\mathrm{II}}}\right)^2 = f_{\mathrm{III}}^2 - \frac{\gamma_{\mathrm{III}}^2\omega_{\mathrm{III}}^2|x_{\mathrm{III}}|^2}{\gamma_{\mathrm{II}}^4}, \tag{76}$$

where $\sigma_{\mathrm{II}} = (\omega_{\text{d-II}} - \omega_{\mathrm{II}})/\gamma_{\mathrm{II}}$ and $\sigma_{\mathrm{III}} = (\omega_{\text{d-III}} - \omega_{\mathrm{III}})/\gamma_{\mathrm{II}}$ are the frequency detunings of the drive

forces, and

$$
\begin{aligned}
M_1 &= -\frac{2\beta_{\mathrm{II}}\beta_{\mathrm{III}}d_0^2}{(4\omega_{\mathrm{II}}^2-\omega_{\mathrm{III}}^2)\gamma_{\mathrm{II}}^2} - \frac{2\beta_{\mathrm{II}}^2d_0^2}{3\omega_{\mathrm{II}}^2\gamma_{\mathrm{II}}^2} - \frac{3\nu_{\mathrm{II}}d_0^2}{\gamma_{\mathrm{II}}^2} \approx -\frac{3\nu_{\mathrm{II}}d_0^2}{\gamma_{\mathrm{II}}^2}, && (77)\\
N_1 &= -\frac{8d_0^2\beta_{\mathrm{II}}}{\gamma_{\mathrm{II}}^2}\left(\frac{\beta_{\mathrm{II}}}{\omega_{\mathrm{III}}^2-4\omega_{\mathrm{II}}^2} + \frac{\beta_{\mathrm{III}}}{\omega_{\mathrm{II}}^2-4\omega_{\mathrm{III}}^2}\right) - \frac{6\nu_{\mathrm{II}}d_0^2}{\gamma_{\mathrm{II}}^2} \approx -\frac{6\nu_{\mathrm{II}}d_0^2}{\gamma_{\mathrm{II}}^2}, && (78)\\
M_2 &= -\frac{2\beta_{\mathrm{II}}\beta_{\mathrm{III}}d_0^2}{(4\omega_{\mathrm{III}}^2-\omega_{\mathrm{II}}^2)\gamma_{\mathrm{II}}^2} - \frac{2\beta_{\mathrm{III}}^2d_0^2}{3\omega_{\mathrm{III}}^2\gamma_{\mathrm{II}}^2} - \frac{3\nu_{\mathrm{III}}d_0^2}{\gamma_{\mathrm{II}}^2} \approx -\frac{3\nu_{\mathrm{III}}d_0^2}{\gamma_{\mathrm{II}}^2}, && (79)\\
N_2 &= -\frac{8d_0^2\beta_{\mathrm{III}}}{\gamma_{\mathrm{II}}^2}\left(\frac{\beta_{\mathrm{II}}}{\omega_{\mathrm{III}}^2-4\omega_{\mathrm{II}}^2} + \frac{\beta_{\mathrm{III}}}{\omega_{\mathrm{II}}^2-4\omega_{\mathrm{III}}^2}\right) - \frac{6\nu_{\mathrm{III}}d_0^2}{\gamma_{\mathrm{II}}^2} \approx -\frac{6\nu_{\mathrm{III}}d_0^2}{\gamma_{\mathrm{II}}^2}, && (80)\\
f_{\mathrm{II}} &= \frac{F_{\mathrm{II}}}{d_0\gamma_{\mathrm{II}}^2}, && (81)\\
f_{\mathrm{III}} &= \frac{F_{\mathrm{III}}}{d_0\gamma_{\mathrm{II}}^2}. && (82)
\end{aligned}
$$

The approximation processes of M_j and N_j $(j = 1,2)$ are based on the facts that $\omega_\square \gg 1$ and $\beta_\square \ll \nu_\square$ $(\square = \mathrm{II}, \mathrm{III})$. Thus, we can conclude that the third-order nonlinearity coefficients are dominant in this system.

The dispersive frequency shift of mode II caused by actuating mode III can be simulated by solving $|x_{\mathrm{II}}|$ using equations (75,76) (Fig.**9**). In this simulation, mode III suffers from stiffness-softening nonlinearity, but it has not been driven into the bifurcation condition.

Based on results depicted in equations (75,76), the frequency shifts of one mode caused by another mode can be explicitly given. We rewrite equations (75,76) into the forms of

$$
\begin{aligned}
\frac{2\omega_{\mathrm{II}}|x_{\mathrm{II}}|\sigma_{\mathrm{II}}}{\gamma_{\mathrm{II}}} &= \pm\sqrt{f_{\mathrm{II}}^2 - \frac{\omega_{\mathrm{II}}^2|x_{\mathrm{II}}|^2}{\gamma_{\mathrm{II}}^2}} - \frac{1}{4}M_1|x_{\mathrm{II}}|^3 - \frac{1}{4}N_1|x_{\mathrm{II}}||x_{\mathrm{III}}|^2, && (83)\\
\frac{2\omega_{\mathrm{III}}|x_{\mathrm{III}}|\sigma_{\mathrm{III}}}{\gamma_{\mathrm{II}}} &= \pm\sqrt{f_{\mathrm{III}}^2 - \frac{\gamma_{\mathrm{III}}^2\omega_{\mathrm{III}}^2|x_{\mathrm{III}}|^2}{\gamma_{\mathrm{II}}^4}} - \frac{1}{4}M_2|x_{\mathrm{III}}|^3 - \frac{1}{4}N_2|x_{\mathrm{II}}|^2|x_{\mathrm{III}}|. && (84)
\end{aligned}
$$

It can be seen in the square roots that the amplitudes of modes II and III should satisfy $|x_{\mathrm{II}}|^2 \le f_{\mathrm{II}}^2\gamma_{\mathrm{II}}^2/\omega_{\mathrm{II}}^2$ and $|x_{\mathrm{III}}|^2 \le f_{\mathrm{III}}^2\gamma_{\mathrm{II}}^4/(\omega_{\mathrm{III}}^2\gamma_{\mathrm{III}}^2)$. When both modes are at resonance, which indicates that amplitudes of both modes reach the maximum, the frequency shifts can be obtained by calculating the detunings σ_{II} and σ_{III} in equations (83,84) and applying conditions $|x_{\mathrm{II}}|^2 = f_{\mathrm{II}}^2\gamma_{\mathrm{II}}^2/\omega_{\mathrm{II}}^2$ and $|x_{\mathrm{III}}|^2 = f_{\mathrm{III}}^2\gamma_{\mathrm{II}}^4/(\omega_{\mathrm{III}}^2\gamma_{\mathrm{III}}^2)$. The frequency shift of mode II $\hat{\sigma}_{\mathrm{II}}$ caused by mode III and that of mode III $\hat{\sigma}_{\mathrm{III}}$ caused by mode II are given by

$$
\begin{aligned}
\hat{\sigma}_{\mathrm{II}} &= -\frac{\gamma_{\mathrm{II}}^3}{8\omega_{\mathrm{II}}}\left[\frac{M_1f_{\mathrm{II}}^2}{\omega_{\mathrm{II}}^2} + \frac{N_1f_{\mathrm{III}}^2\gamma_{\mathrm{II}}^2}{\omega_{\mathrm{III}}^2\gamma_{\mathrm{III}}^2}\right] \approx \frac{3\nu_{\mathrm{II}}d_0^2\gamma_{\mathrm{II}}}{8\omega_{\mathrm{II}}}\left[\frac{f_{\mathrm{II}}^2}{\omega_{\mathrm{II}}^2} + \frac{2f_{\mathrm{III}}^2\gamma_{\mathrm{II}}^2}{\omega_{\mathrm{III}}^2\gamma_{\mathrm{III}}^2}\right], && (85)\\
\hat{\sigma}_{\mathrm{III}} &= -\frac{\gamma_{\mathrm{II}}^3}{8\omega_{\mathrm{III}}}\left[\frac{M_2f_{\mathrm{III}}^2\gamma_{\mathrm{II}}^2}{\omega_{\mathrm{III}}^2\gamma_{\mathrm{III}}^2} + \frac{N_2f_{\mathrm{II}}^2}{\omega_{\mathrm{II}}^2}\right] \approx \frac{3\nu_{\mathrm{III}}d_0^2\gamma_{\mathrm{II}}}{8\omega_{\mathrm{III}}}\left[\frac{f_{\mathrm{III}}^2\gamma_{\mathrm{II}}^2}{\omega_{\mathrm{III}}^2\gamma_{\mathrm{III}}^2} + \frac{2f_{\mathrm{II}}^2}{\omega_{\mathrm{II}}^2}\right]. && (86)
\end{aligned}
$$

The sign (direction) of the frequency shift is determined by that of the third-order nonlinearity coefficients ν_{II} and ν_{III}. In fact, the experimentally observed frequency shift of mode II caused by actuation of mode III is mostly caused by the $3\nu_{\mathrm{II}}x_{\mathrm{III}}^2x_{\mathrm{II}}$ term in expanded equation (73).

Supplementary Note 5. Coupling-abundant multiple-mode system

The experimentally observed skewed "#" configuration can be simulated by separately modeling the sequential dynamical coupling of III-1 to II-1 and II-2 and that of III-2 to II-1 and II-2. Here, we give the theoretical model for the sequential dynamical coupling of one higher mode III (refering to III-1 or III-2) to order-two modes. The drive and pump signals are applied along H-1 and H-2 hybrid states, respectively. The higher mode III is used as the phonon cavity. Before the drive and pump are applied. The initial electrical potential energy is given by.

$$U_{\mathrm{e0}} = -\frac{A_{\mathrm{d}}\epsilon_0 V_0^2}{2(d_0 + x + z)} - \frac{A_{\mathrm{p}}\epsilon_0 V_0^2}{2(d_0 + y + z)}, \tag{87}$$

where, x, y, and z are the displacements of hybrid states H-1, H-2, and mode III, respectively. This initial electrical potential energy is caused by the bias V_0 applied on the resonator body, which results in the frequency shift for the mechanical resonator. We'd rather include this static potential energy as part of the initial mechanical potential energy. Thus, the potential energy of the system when drive and pump are applied is given by

$$\begin{aligned} U =& \frac{k_{\mathrm{II\text{-}1}}x_\omega^2}{2} + \frac{k_{\mathrm{II\text{-}2}}y_\omega^2}{2} + \frac{k_{\mathrm{III}}z^2}{2} - \frac{A_{\mathrm{d}}\epsilon_0[V_{\mathrm{d}}\cos(\omega_{\mathrm{d}}t) - V_0]^2 - A_{\mathrm{d}}\epsilon_0 V_0^2}{2(d_0 + x + z)} \\ &- \frac{A_{\mathrm{p}}\epsilon_0[V_{\mathrm{p}}\cos(\omega_{\mathrm{p}}t) - V_0]^2 - A_{\mathrm{p}}\epsilon_0 V_0^2}{2(d_0 + y + z)}, \end{aligned} \tag{88}$$

where $k_\square$ ($\square$ =II-1, II-2, III) is the effective stiffness of normal modes II-1, II-2, and III, in which the initial electrical potential energy induced stiffness change has been considered. The displacements x, y of hybrid states H-1, H-2 can be transformed into displacements x_ω, y_ω of normal modes II-1, II-2 using coordinate-transformation matrix $\mathbf{p}$. By using Lagrange method, the equations of motion can

be obtained as

$$m_{\text{II-1}}\ddot{x}_\omega + k_{\text{II-1}}x_\omega + \frac{\text{DC}_1\cos\theta}{4(d_0 + x_\omega\cos\theta - y_\omega\sin\theta + z)^2} - \frac{\text{AC}_1\cos\theta\cos(\omega_\text{d}t)}{(d_0 + x_\omega\cos\theta - y_\omega\sin\theta + z)^2}$$
$$+ \frac{\text{DC}_2\sin\theta}{4(d_0 + x_\omega\sin\theta + y_\omega\cos\theta + z)^2} - \frac{\text{AC}_2\sin\theta\cos(\omega_\text{p}t)}{(d_0 + x_\omega\sin\theta + y_\omega\cos\theta + z)^2} = 0, \tag{89}$$

$$m_{\text{II-2}}\ddot{y}_\omega + k_{\text{II-2}}y_\omega - \frac{\text{DC}_1\sin\theta}{4(d_0 + x_\omega\cos\theta - y_\omega\sin\theta + z)^2} + \frac{\text{AC}_1\sin\theta\cos(\omega_\text{d}t)}{(d_0 + x_\omega\cos\theta - y_\omega\sin\theta + z)^2}$$
$$+ \frac{\text{DC}_2\cos\theta}{4(d_0 + x_\omega\sin\theta + y_\omega\cos\theta + z)^2} - \frac{\text{AC}_2\cos\theta\cos(\omega_\text{p}t)}{(d_0 + x_\omega\sin\theta + y_\omega\cos\theta + z)^2} = 0, \tag{90}$$

$$m_{\text{III}}\ddot{z} + k_{\text{III}}z + \frac{\text{DC}_1}{4(d_0 + x_\omega\cos\theta - y_\omega\sin\theta + z)^2} - \frac{\text{AC}_1\cos(\omega_\text{d}t)}{(d_0 + x_\omega\cos\theta - y_\omega\sin\theta + z)^2}$$
$$+ \frac{\text{DC}_2}{4(d_0 + x_\omega\sin\theta + y_\omega\cos\theta + z)^2} - \frac{\text{AC}_2\cos(\omega_\text{p}t)}{(d_0 + x_\omega\sin\theta + y_\omega\cos\theta + z)^2} = 0. \tag{91}$$

Here,

$$\text{DC}_1 = A_\text{d}\epsilon_0 V_\text{d}^2, \tag{92}$$

$$\text{AC}_1 = A_\text{d}\epsilon_0 V_0 V_\text{d}, \tag{93}$$

$$\text{DC}_2 = A_\text{p}\epsilon_0 V_\text{p}^2, \tag{94}$$

$$\text{AC}_2 = A_\text{p}\epsilon_0 V_0 V_\text{p}. \tag{95}$$

Expanding the nonlinear restoring forces into Taylor series respect to x_ω, y_ω, and z, and neglecting higher order terms, we obtain

$$m_{\text{II-1}}\ddot{x}_\omega + k_{\text{II-1}}x_\omega + \left[\frac{\text{DC}_1\cos\theta}{4} - \text{AC}_1\cos\theta\cos(\omega_\text{d}t)\right]\left\{\frac{1}{d_1^2} - \frac{2}{d_1^3}\big[(x_\omega - x_{\omega,0})\cos\theta\right.$$
$$\left. - (y_\omega - y_{\omega,0})\sin\theta + (z - z_0)\big]\right\} + \left[\frac{\text{DC}_2\sin\theta}{4} - \text{AC}_2\sin\theta\cos(\omega_\text{p}t)\right]\left\{\frac{1}{d_2^2}\right.$$
$$\left. - \frac{2}{d_2^3}\big[(x_\omega - x_{\omega,0})\sin\theta + (y_\omega - y_{\omega,0})\cos\theta + (z - z_0)\big]\right\}, \tag{96}$$

$$m_{\text{II-2}}\ddot{y}_\omega + k_{\text{II-2}}y_\omega + \left[-\frac{\text{DC}_1\sin\theta}{4} + \text{AC}_1\sin\theta\cos(\omega_\text{d}t)\right]\left\{\frac{1}{d_1^2} - \frac{2}{d_1^3}\big[(x_\omega - x_{\omega,0})\cos\theta\right.$$
$$\left. - (y_\omega - y_{\omega,0})\sin\theta + (z - z_0)\big]\right\} + \left[\frac{\text{DC}_2\cos\theta}{4} - \text{AC}_2\cos\theta\cos(\omega_\text{p}t)\right]\left\{\frac{1}{d_2^2}\right.$$
$$\left. - \frac{2}{d_2^3}\big[(x_\omega - x_{\omega,0})\sin\theta + (y_\omega - y_{\omega,0})\cos\theta + (z - z_0)\big]\right\}, \tag{97}$$

$$m_{\text{III}}\ddot{z} + k_{\text{III}}z + \left[\frac{\text{DC}_1}{4} - \text{AC}_1\cos(\omega_\text{d}t)\right]\left\{\frac{1}{d_1^2} - \frac{2}{d_1^3}\big[(x_\omega - x_{\omega,0})\cos\theta\right.$$
$$\left. - (y_\omega - y_{\omega,0})\sin\theta + (z - z_0)\big]\right\} + \left[\frac{\text{DC}_2}{4} - \text{AC}_2\cos(\omega_\text{p}t)\right]\left\{\frac{1}{d_2^2}\right.$$
$$\left. - \frac{2}{d_2^3}\big[(x_\omega - x_{\omega,0})\sin\theta + (y_\omega - y_{\omega,0})\cos\theta + (z - z_0)\big]\right\}. \tag{98}$$

Here, $d_1 = d_0 + x_{\omega,0}\cos\theta - y_{\omega,0}\sin\theta + z_0$ and $d_2 = d_0 + x_{\omega,0}\sin\theta + y_{\omega,0}\cos\theta + z_0$. $x_{\omega,0}$, $y_{\omega,0}$, and z_0 are new equilibrium positions, which satisfy

$$\frac{\mathrm{DC}_1\cos\theta}{4d_1^2} + \frac{\mathrm{DC}_2\sin\theta}{4d_2^2} + k_{\text{II-1}}x_{\omega,0} = 0, \tag{99}$$

$$\frac{\mathrm{DC}_2\cos\theta}{4d_2^2} - \frac{\mathrm{DC}_1\sin\theta}{4d_1^2} + k_{\text{II-2}}y_{\omega,0} = 0, \tag{100}$$

$$\frac{\mathrm{DC}_1}{4d_1^2} + \frac{\mathrm{DC}_2}{4d_2^2} + k_{\text{III}}z_0 = 0. \tag{101}$$

Denoting $u = x_\omega - x_{\omega,0}$, $v = y_\omega - y_{\omega,0}$, and $w = z - z_0$, the equations of motion can be rewritten as

$$\begin{aligned}
&m_{\text{II-1}}\ddot{u} + k_{\text{II-1}}u - \left(\frac{\mathrm{DC}_1}{2d_1^3}\cos^2\theta + \frac{\mathrm{DC}_2}{2d_2^3}\sin^2\theta\right)u + \left(\frac{\mathrm{DC}_1}{2d_1^3}\cos\theta\sin\theta - \frac{\mathrm{DC}_2}{2d_2^3}\cos\theta\sin\theta\right)v \\
&- \left(\frac{\mathrm{DC}_1}{2d_1^3}\cos\theta + \frac{\mathrm{DC}_2}{2d_2^3}\sin\theta\right)w - \frac{\mathrm{AC}_1}{d_1^2}\cos\theta\cos(\omega_\mathrm{d}t) - \frac{\mathrm{AC}_2}{d_2^2}\sin\theta\cos(\omega_\mathrm{p}t) \\
&+ \frac{2\mathrm{AC}_1}{d_1^3}\cos\theta\cos(\omega_\mathrm{d}t)(u\cos\theta - v\sin\theta + w) \\
&+ \frac{2\mathrm{AC}_2}{d_2^3}\sin\theta\cos(\omega_\mathrm{p}t)(u\sin\theta + v\cos\theta + w) = 0,
\end{aligned} \tag{102}$$

$$\begin{aligned}
&m_{\text{II-2}}\ddot{v} + k_{\text{II-2}}v + \left(\frac{\mathrm{DC}_1}{2d_1^3}\cos\theta\sin\theta - \frac{\mathrm{DC}_2}{2d_2^3}\cos\theta\sin\theta\right)u - \left(\frac{\mathrm{DC}_1}{2d_1^3}\sin^2\theta + \frac{\mathrm{DC}_2}{2d_2^3}\cos^2\theta\right)v \\
&+ \left(\frac{\mathrm{DC}_1}{2d_1^3}\sin\theta - \frac{\mathrm{DC}_2}{2d_2^3}\cos\theta\right)w + \frac{\mathrm{AC}_1}{d_1^2}\sin\theta\cos(\omega_\mathrm{d}t) - \frac{\mathrm{AC}_2}{d_2^2}\cos\theta\cos(\omega_\mathrm{p}t) \\
&- \frac{2\mathrm{AC}_1}{d_1^3}\sin\theta\cos(\omega_\mathrm{d}t)(u\cos\theta - v\sin\theta + w) \\
&+ \frac{2\mathrm{AC}_2}{d_2^3}\cos\theta\cos(\omega_\mathrm{p}t)(u\sin\theta + v\cos\theta + w) = 0,
\end{aligned} \tag{103}$$

$$\begin{aligned}
&m_{\text{III}}\ddot{w} + k_{\text{III}}w - \left(\frac{\mathrm{DC}_1}{2d_1^3}\cos\theta + \frac{\mathrm{DC}_2}{2d_2^3}\sin\theta\right)u + \left(\frac{\mathrm{DC}_1}{2d_1^3}\sin\theta - \frac{\mathrm{DC}_2}{2d_2^3}\cos\theta\right)v \\
&- \left(\frac{\mathrm{DC}_1}{2d_1^3} + \frac{\mathrm{DC}_2}{2d_2^3}\right)w - \frac{\mathrm{AC}_1}{d_1^2}\cos(\omega_\mathrm{d}t) - \frac{\mathrm{AC}_2}{d_2^2}\cos(\omega_\mathrm{p}t) \\
&+ \frac{2\mathrm{AC}_1}{d_1^3}\cos(\omega_\mathrm{d}t)(u\cos\theta - v\sin\theta + w) \\
&+ \frac{2\mathrm{AC}_2}{d_2^3}\cos(\omega_\mathrm{p}t)(u\sin\theta + v\cos\theta + w) = 0.
\end{aligned} \tag{104}$$

Neglecting the non-resonance driving and pumping terms, and additionally introducing damping

terms, the EOMs can be simplified as

$$\ddot{u} + \gamma_{\text{II}}\dot{u} + \omega_{\text{II-1}}^2 u + \alpha_1 u + \beta_1 v + \lambda_1 w + \Lambda_1 \cos(\omega_{\text{p}} t)(u \sin\theta + v \cos\theta + w) = g_1 \cos(\omega_{\text{d}} t), \tag{105}$$

$$\ddot{v} + \gamma_{\text{II}}\dot{v} + \omega_{\text{II-2}}^2 v + \alpha_2 u + \beta_2 v + \lambda_2 w + \Lambda_2 \cos(\omega_{\text{p}} t)(u \sin\theta + v \cos\theta + w) = g_2 \cos(\omega_{\text{d}} t), \tag{106}$$

$$\ddot{w} + \gamma_{\text{III}}\dot{w} + \omega_{\text{III}}^2 w + \alpha_3 u + \beta_3 v + \lambda_3 w + \Lambda_3 \cos(\omega_{\text{p}} t)(u \sin\theta + v \cos\theta + w) = 0, \tag{107}$$

where

$$\alpha_1 = -\frac{\text{DC}_1}{2d_1^3 m_{\text{II}}}\cos^2\theta - \frac{\text{DC}_2}{2d_2^3 m_{\text{II}}}\sin^2\theta, \tag{108}$$

$$\beta_1 = \frac{\text{DC}_1}{2d_1^3 m_{\text{II}}}\cos\theta\sin\theta - \frac{\text{DC}_2}{2d_2^3 m_{\text{II}}}\cos\theta\sin\theta, \tag{109}$$

$$\lambda_1 = -\frac{\text{DC}_1}{2d_1^3 m_{\text{II}}}\cos\theta - \frac{\text{DC}_2}{2d_2^3 m_{\text{II}}}\sin\theta, \tag{110}$$

$$\Lambda_1 = \frac{2\text{AC}_2}{d_2^3 m_{\text{II}}}\sin\theta, \tag{111}$$

$$g_1 = \frac{\text{AC}_1}{d_1^2 m_{\text{II}}}\cos\theta, \tag{112}$$

$$\alpha_2 = \frac{\text{DC}_1}{2d_1^3 m_{\text{II}}}\cos\theta\sin\theta - \frac{\text{DC}_2}{2d_2^3 m_{\text{II}}}\cos\theta\sin\theta, \tag{113}$$

$$\beta_2 = -\frac{\text{DC}_1}{2d_1^3 m_{\text{II}}}\sin^2\theta - \frac{\text{DC}_2}{2d_2^3 m_{\text{II}}}\cos^2\theta, \tag{114}$$

$$\lambda_2 = \frac{\text{DC}_1}{2d_1^3 m_{\text{II}}}\sin\theta - \frac{\text{DC}_2}{2d_2^3 m_{\text{II}}}\cos\theta, \tag{115}$$

$$\Lambda_2 = \frac{2\text{AC}_2}{d_2^3 m_{\text{II}}}\cos\theta, \tag{116}$$

$$g_2 = -\frac{\text{AC}_1}{d_1^2 m_{\text{II}}}\sin\theta, \tag{117}$$

$$\alpha_3 = -\frac{\text{DC}_1}{2d_1^3 m_{\text{III}}}\cos\theta - \frac{\text{DC}_2}{2d_2^3 m_{\text{III}}}\sin\theta, \tag{118}$$

$$\beta_3 = \frac{\text{DC}_1}{2d_1^3 m_{\text{III}}}\sin\theta - \frac{\text{DC}_2}{2d_2^3 m_{\text{III}}}\cos\theta, \tag{119}$$

$$\lambda_3 = -\frac{\text{DC}_1}{2d_1^3 m_{\text{III}}} - \frac{\text{DC}_2}{2d_2^3 m_{\text{III}}}, \tag{120}$$

$$\Lambda_3 = \frac{2\text{AC}_2}{d_2^3 m_{\text{III}}}. \tag{121}$$

Equations (105,106,107) can be solved by using rotating-frame-approximation with higher-idler

resonances. Suppose the solutions of (105,106,107) can be written as

$$u = \sum_{m=-\infty}^{+\infty} \Big[a_m(t)e^{i(\omega_\mathrm{d}+m\omega_\mathrm{p})t} + cc\Big], \tag{122}$$

$$v = \sum_{m=-\infty}^{+\infty} \Big[b_m(t)e^{i(\omega_\mathrm{d}+m\omega_\mathrm{p})t} + cc\Big], \tag{123}$$

$$w = \sum_{m=-\infty}^{+\infty} \Big[c_m(t)e^{i(\omega_\mathrm{d}+m\omega_\mathrm{p})t} + cc\Big], \tag{124}$$

where a_m, b_m, and c_m are slowly varying complex amplitudes of the m-th order idler resonance, and m is any integer. Substituting them and their first- and second-order derivative respect to time into equations (105,106,107), we obtain

$$\begin{aligned}
&\big[2i(\omega_\mathrm{d}+m\omega_\mathrm{p})+\gamma_\mathrm{II}\big]\dot{a}_m + \Big[-(\omega_\mathrm{d}+m\omega_\mathrm{p})^2 + i\gamma_\mathrm{II}(\omega_\mathrm{d}+m\omega_\mathrm{p}) + \omega_\text{II-1}^2 + \alpha_1\Big]a_m + \beta_1 b_m \\
&+ \lambda_1 c_m + \frac{\Lambda_1}{2}(a_{m-1}\sin\theta + b_{m-1}\cos\theta + c_{m-1}) + \frac{\Lambda_1}{2}(a_{m+1}\sin\theta + b_{m+1}\cos\theta + c_{m+1}) \\
&= \frac{g_1}{2}\delta(m),
\end{aligned} \tag{125}$$

$$\begin{aligned}
&\big[2i(\omega_\mathrm{d}+m\omega_\mathrm{p})+\gamma_\mathrm{II}\big]\dot{b}_m + \Big[-(\omega_\mathrm{d}+m\omega_\mathrm{p})^2 + i\gamma_\mathrm{II}(\omega_\mathrm{d}+m\omega_\mathrm{p}) + \omega_\text{II-2}^2 + \beta_2\Big]b_m + \alpha_2 a_m \\
&+ \lambda_2 c_m + \frac{\Lambda_2}{2}(a_{m-1}\sin\theta + b_{m-1}\cos\theta + c_{m-1}) + \frac{\Lambda_2}{2}(a_{m+1}\sin\theta + b_{m+1}\cos\theta + c_{m+1}) \\
&= \frac{g_1}{2}\delta(m),
\end{aligned} \tag{126}$$

$$\begin{aligned}
&\big[2i(\omega_\mathrm{d}+m\omega_\mathrm{p})+\gamma_\mathrm{III}\big]\dot{c}_m + \Big[-(\omega_\mathrm{d}+m\omega_\mathrm{p})^2 + i\gamma_\mathrm{III}(\omega_\mathrm{d}+m\omega_\mathrm{p}) + \omega_\mathrm{III}^2 + \lambda_3\Big]c_m + \alpha_3 a_m \\
&+ \beta_3 b_m + \frac{\Lambda_3}{2}(a_{m-1}\sin\theta + b_{m-1}\cos\theta + c_{m-1}) + \frac{\Lambda_3}{2}(a_{m+1}\sin\theta + b_{m+1}\cos\theta + c_{m+1}) \\
&= 0.
\end{aligned} \tag{127}$$

The second time derivatives of the complex amplitudes have been neglected. The frequency responses of normal modes can be obtained by applying steady-state condition $\dot{a}_m = \dot{b}_m = \dot{c}_m = 0$ and calculating $|a_0|$ and $|b_0|$. The experimental frequency response of the modes II can be reproduced by transforming amplitudes of $x_\omega - o - y_\omega$ to $x - o - y$ using coordinate-transformation matrix $\mathbf{p}$. Be simulating the coupling of modes III-1 to II-1 and II-2, the lower half of the skewed "#" in Figure 4g is obtained. Likewise, the higher half of the skewed "#" can also be obtained by simulating the coupling of modes III-2 to II-1 and II-2.

Supplementary References

[1] Y. Yang, E. J. Ng, Y. Chen, I. B. Flader, and T. W. Kenny, "A unified Epi-seal process for fabrication of high-stability microelectromechanical devices," Journal of Microelectromechanical Systems **25**, 489–497 (2016).

[2] M. Frimmer and L. Novotny, "The classical Bloch equations," American Journal of Physics **82**, 947–954 (2014).

[3] H. Okamoto, A. Gourgout, C.-Y. Chang, K. Onomitsu, I. Mahboob, E. Y. Chang, and H. Yamaguchi, "Coherent phonon manipulation in coupled mechanical resonators," Nature Physics **9**, 598–598 (2013).

[4] A. H. Nayfeh and D. T. Mook, *Nonlinear Oscillations* (Wiley-VCH Verlag GmbH & Co. KGaA, 1995).
